# Supplementary material for: Two Late Cretaceous sauropods reveal titanosaurian dispersal across South America
Source: Commun Biol. 2020 Oct 27;3:622. doi: 10.1038/s42003-020-01338-w (PMC7591563; doi:10.1038/s42003-020-01338-w)
Supplement: Supplementary file 1 — Supplementary Information [file 42003_2020_1338_MOESM1_ESM.pdf]

ELECTRONIC SUPPLEMENTARY INFORMATION

for

Two Late Cretaceous sauropods reveal titanosaurian dispersal across South America

E. Martín Hechenleitner*, Léa Leuzinger, Agustín G. Martinelli, Sebastián Rocher, Lucas E. Fiorelli, Jeremías R.A. Taborda, Leonardo Salgado

*Corresponding author emails: martinh@conicet.gov.ar; emhechenleitner@gmail.com

This file includes:

Geological setting	2
Taphonomy	6
Measurements	9
Titanosauria in South America	12
Anatomical characters	14
Phylogenetic analysis	16
Latitudinal position of South American taxa	20
Character list	22
References	53

Geological setting

The Andean Precordillera of western Argentina (Supplementary Fig. 1a) preserves a protracted geological history of convergence and accretion that took place along the south-western margin of Gondwana since at least the early Palaeozoic (Ramos, 1988). The present relief of Precordillera results from crustal thickening that occurred mainly in Neogene times during the Andean orogeny (Jordan et al., 1983). Contractional deformation resulted in a fold-and-thrust belt that exposed Palaeozoic, Mesozoic and Cenozoic strata (Bracaccini, 1946).

Since the 1990s, paleontological discoveries have allowed sedimentary units in the northern Precordillera to be ascribed an Upper Cretaceous age. Main outcrop areas with published stratigraphic studies are Huaco (Fosdick et al., 2017; Limarino et al., 2000; Reat and Fosdick, 2018), Puesto La Flecha (Ciccioli et al., 2005), and Río La Troya (Tedesco et al., 2007) (Supplementary Fig. 1a). These strata consist of a 100-200 m thick red bed succession deposited in fluvial and lacustrine environments referred to collectively as the Ciénaga del Río Huaco Formation (Limarino et al., 2000). The previously known fossil record includes palynomorphs and freshwater ostracods (Chaía, 1990; Ciccioli et al., 2005; Limarino et al., 2000; Pérez et al., 1993). The microfossil assemblages allowed an Upper Cretaceous age (Maastrichtian) to be inferred for the upper part of the unit. Additionally, a radiometric age of 108.1 ± 4.4 Ma (Albian) by K-Ar method in a tuff was determined by Tedesco et al. (2007) in La Troya section (Supplementary Fig. 1a). More recently Fosdick et al. (2017) and Reat and Fosdick (2018) established a maximum depositional age of ~96-93 Ma (Cenomanian-Turonian) based on interpretation of detrital zircon U-Pb ages on samples from the lower part of the unit at the Huaco area. The combined biostratigraphic and geochronological data suggest a wide time range for the deposition of the Ciénaga del Río Huaco Formation within the Late Cretaceous and highlight the lack of more precise absolute ages to establish an accurate chronostratigraphic framework for the unit.

Outcrops of Ciénaga del Río Huaco Formation were recognized in subsequent studies at Quebrada Santo Domingo (QSD), a locality of the Andean Precordillera of La Rioja province (Supplementary Fig. 1a). For this area, Arcucci et al. (2005) and Hechenleitner et al. (2018) reported fragmentary caudal remains of a titanosaurian sauropod collected by the geologists Tim Coughlin and Rod Holcombe (Coughlin,

2000) and ascribed the fossil-bearing rocks to the Ciénaga del Río Huaco Formation. A stratigraphic description and map of the unit were recently offered by Limarino et al. (2016).

At QSD area, the Ciénaga del Río Huaco Formation consists of a sandy-silty succession with scarce conglomerates (Supplementary Fig. 1b). It disconformably overlies the Upper Triassic Santo Domingo Formation and supports, in apparent transition, the Eocene Puesto La Flecha Formation (Supplementary Fig. 1c). In our stratigraphic inspections, we note that sandstone sheets interleaved with siltstones and nodular gypsum of the upper part of Ciénaga del Río Huaco Formation are gradually replaced upwards by laminated siltstones and fine sandstones and higher up to fine to medium sandstones that characterize the lower Puesto La Flecha Formation. We do not discard that this transition could be stratigraphically representative of the Puesto La Flecha Formation as it was considered by Fosdick et al. (2017) and Reat and Fosdick (2018). In this scenario, *Punatitan* would belong to the basal portion of the Puesto La Flecha Formation. For the lower part of this unit Reat and Fosdick (2018) determined a ~65 Ma maximum depositional age. According to the interpretation of the authors, this transition can represent a low accommodation depocentre with lacustrine sedimentation during Palaeogene times containing numerous disconformities. To avoid confusion, we adopt the name of Ciénaga del Río Huaco Formation for the stratigraphic interval containing dinosaur remains. We place the limit between both units 250 m above the base of Ciénaga del Río Huaco Formation which is the thickness ascribed for this unit at Quebrada Santo Domingo (Limarino et al., 2016).

Here we divided the Ciénaga del Río Huaco Formation strata at QSD into lower, middle and upper sections.

Lower section. The lower section is a 103 m thick interval of moderate reddish-brown colour formed by regular intercalations of sandstone bodies interleaved between siltstones (Supplementary Fig. 1b, c). Conglomeratic facies are infrequent and mostly present in the basal section in close association with sandy facies. They occur as the infill of narrow and relatively deep channels and are interpreted as bottom channel fills and gravelly bedforms. The sandstones commonly show well-developed internal structure characterized by trough and planar cross-bedding, although structureless sandstones also occur. Basal contacts are mostly defined by low angle erosive scours.

Sandstones represent various migrating bedforms deposited in lower, transitional and upper flow regimes. They are present mainly as channel fills and proximal and distal overbank deposits (levee and crevasse splay deposits). Siltstones occur in thick intervals that host thin intercalations of sandstone sheets. They show massive or horizontal lamination structure. These fine-grained rocks represent proximal overbank deposits accumulated in floodplains, from suspension load. In association with the overbank fines, some thin tabular layers of limestone occur. The presence of limestone is here interpreted as the resultant of chemically or biochemically induced precipitation of carbonates in small saline ephemeral lakes or ponds. Pedogenetic alterations were also recognized in some horizons of the overbank fine deposits mainly present in the form of root marks, as well as calcite and silica nodules. Facies and architectural elements described here suggest a paleoenvironment characterized by the development of fine-grained, mixed-load meandering rivers (Bridge, 2003; Miall, 1996).

Middle section. The middle section is a 24 m thick interval formed mostly by trough cross-bedded coarse sandstones that can be easily recognized by its conspicuous light greyish color (Supplementary Fig. 1b, c). The unit shows variations including moderate to poorly sorted, coarse, very coarse and some clast-supported pebble conglomerate lenses. They form 2-5 m thick channelized bodies with scoured bases, and sparse pebble basal lags that fine upward into well-developed, large-scale, trough-cross bedded, coarse- and medium-grained sandstones. The facies are interpreted as bottom channel fills and sandy bedforms. This interval represents deposits of a sand-bed, braided fluvial system with high energy transport (Allen, 1983; Miall, 1996).

Upper section. The upper section is a 116 m thick moderate red coloured silty-sandy interval (Supplementary Fig. 1b, c). The lower part consists of tabular beds of regularly to well-sorted medium- to fine-grained sandstones, usually graded, massive or planar cross-bedded, interleaved with massive and fine laminated siltstones and mudstones, including sparse nodular gypsum and thin beds of marls. The lower thick sandstones represent deposits of sheet-type flood surges. High evaporative rates produced chemical precipitation of evaporite minerals. Upsection, deposits are gradually replaced by finely laminated siltstone and mudstone with sparse intercalations of thin sheets of fine sandstones. Fine sediments settled during relatively prolonged periods in temporary water bodies, which were occasionally interrupted by the invasion of flood surges that resulted in spreading of fine sand blankets. The lower and upper parts of this section

represent respectively sand to mudflats of the margins and floor of an ephemeral lake system (Tunbridge, 1984).

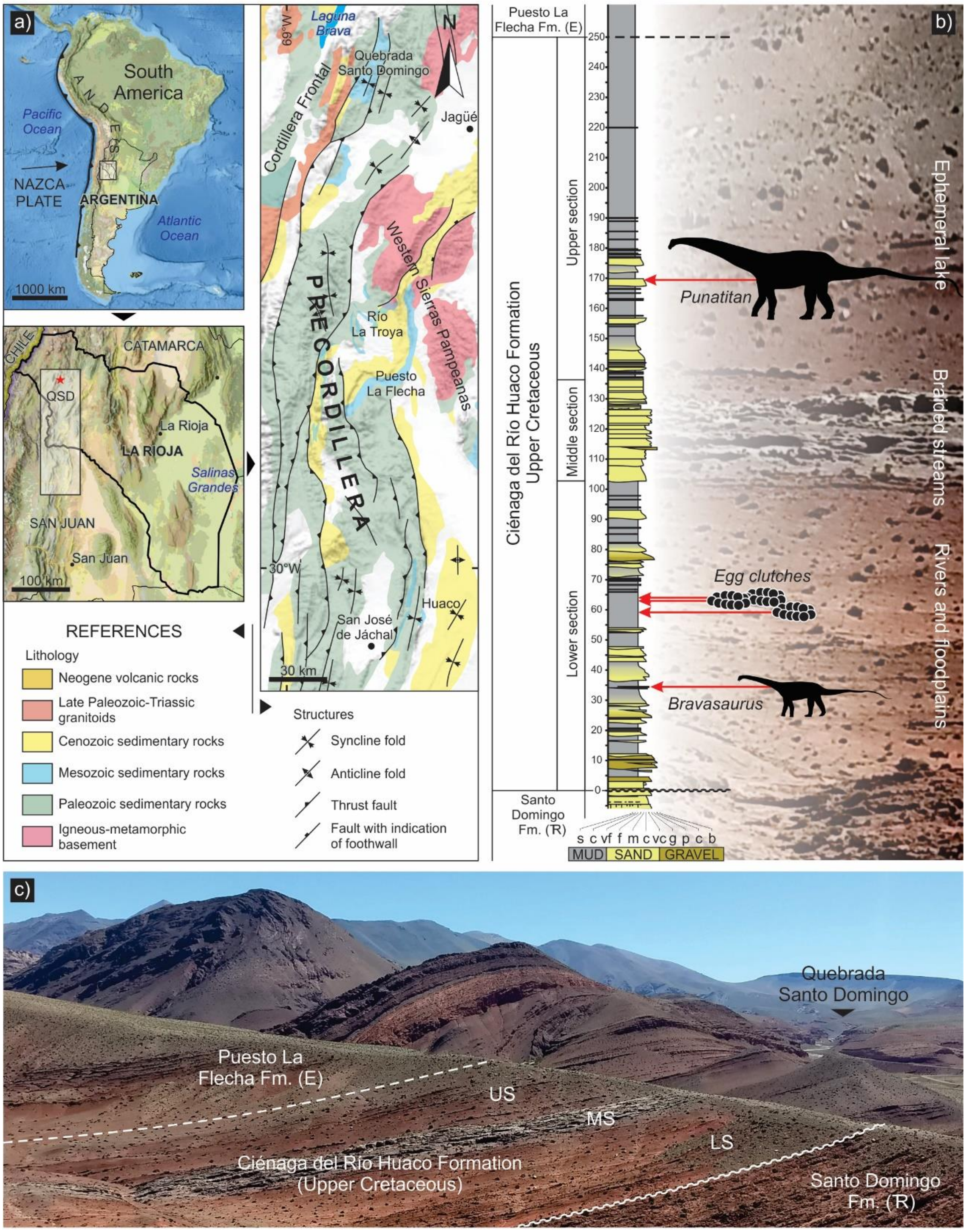

Supplementary Fig. 1. Geographical and geological settings of the Ciénaga del Río Huaco Formation at QSD. a Relative location of the studied area in northern Precordillera, La Rioja province, NW Argentina. It shows the location of regions with stratigraphic studies: Huaco, Puesto La Flecha and Río La Troya. **b** Stratigraphic section with an indication of fossil-bearing levels. **c** Panoramic photograph of QSD area showing field relations of Ciénaga del Río Huaco Formation. The column is in metres. LS: lower section; MS: middle section; US: upper section.

Taphonomy

Partial skeletons and egg clutches are found in different horizons of the Ciénaga del Río Huaco Formation at QSD. They differ in their depositional modes and preservation.

Bravasaurus. The remains of *Bravasaurus*, both the holotype and the paratype, consist of partial skeletons found at the lower section of the Ciénaga del Río Huaco Formation, approximately at the same horizon, 34 m above the base of the unit (Supplementary Fig. 1b), but separated laterally over a distance of 240 m. In both cases, they occur in 0.5 to 2 m thick beds of moderately to poorly sorted medium-grained sandstone, mostly structureless or with diffuse horizontal and cross-beddings. These features and association with floodplain deposits are interpreted as characteristic of proximal crevasse splay deposits (Burns et al., 2017). Bones were found semi-articulated with no evidence of abrasion and weathering. Long bones in this layer tend to have their long axes oriented NW-SE (Supplementary Fig. 2). These features suggest fortuitous preservation of dead bodies with a short exposure and only partial disarticulation. The remains had minimal transport and were quickly buried during events with a high rate of deposition such as channel overbanks that produce crevasse splays (Behrensmeyer and Hook, 1992).

Punatitan. The remains of *Punatitan* consist of a partial skeleton found at the upper section of the Ciénaga del Río Huaco Formation, 170 m above the base of the unit (Supplementary Fig. 1b). The deposit that contains the fossils is a 2.2 m thick graded bed of medium- to fine-grained sandstone. Its basal part shows a horizontal and planar cross-bedded arrangement, whereas the upper portion is structureless. It is interpreted as a sandy lobe deposit, part of the marginal sand flat of an ephemeral lake system (Hubert and Hyde, 1982). The remains are semi-articulated, closely spaced and partly stacked on each other with no evidence of abrasion. Long elements of the skeleton are disposed of with a preferred NE-SW orientation (Supplementary Fig. 3). The good preservation and sedimentological features suggest rapid deposition of sand and quick burial, preventing higher degrees of disaggregation and dispersion (Behrensmeyer and Hook, 1992). Favourable conditions are created during flash flood events that spread out in sheet flows, decelerating and promoting high deposition rates. The sheet-like progradational mode of deposition probably favoured minimal transport and staking of the remains.

Titanosaurian eggs. Eggshells and egg clutches were found in at least three distinct but closely spaced horizons in a 5 m thick interval located 59-64 m above the base of the Ciénaga del Río Huaco Formation (Supplementary Fig. 1b). This interval can be traced laterally for more than 3 km. The deposits consist of laminated siltstones and mudstones commonly in mixture with very fine sand-sized grains. Lamination is recognized by subtle centimetre-scale variations in grain size, between silt and clay, and by different proportions of fine sand. They represent both rapid and gradual deposition from a suspensive load of low-energy flow during flooding events in a floodplain setting. The bedding was caused by variations in flood energy during deposition and different flooding events. Particularly, eggs in clutches occur closely packed and show one- or two-row arrangement in cross-section. The latter occupy a vertical space within the strata of ~15-30 cm, which means that they are contained by various flooding events, each one typically a few centimetres thick. Since it is unlikely that spherical, low-density objects, stacked on a flatbed (the floodplain), may be able to stay grouped *in situ* during and between several flood events, titanosaurians must have adopted a burial nesting strategy (Fowler and Hall, 2011; Hechenleitner et al., 2015; Vila et al., 2010a, 2010b) that would have prevented the collapse and transport of eggs. The low-energy floodplain depositional setting and the proximity to water bodies would be preferred places for nesting.

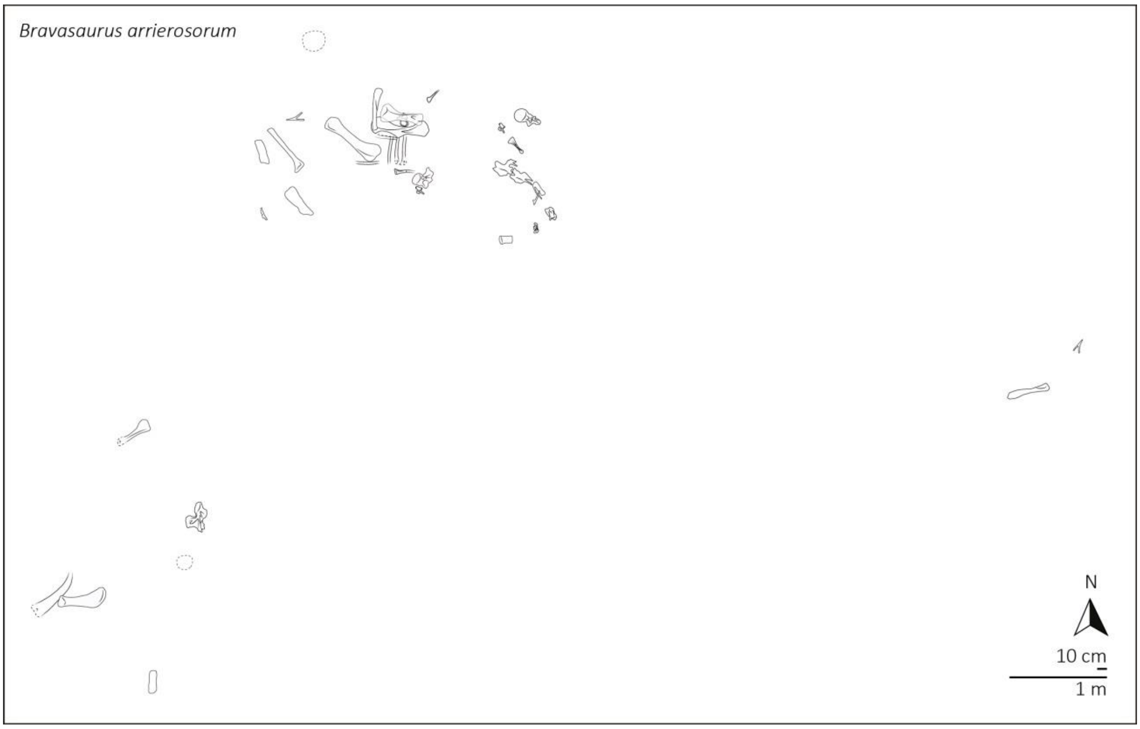

Supplementary Fig. 2. Outcrop map of the *Bravasaurus* gen. nov. holotype quarry. Distances between skeletal elements and their respective orientations were restored to their original horizontal position, as the bearing strata dip c. 30º. Dotted lines represent undetermined bone fragments.

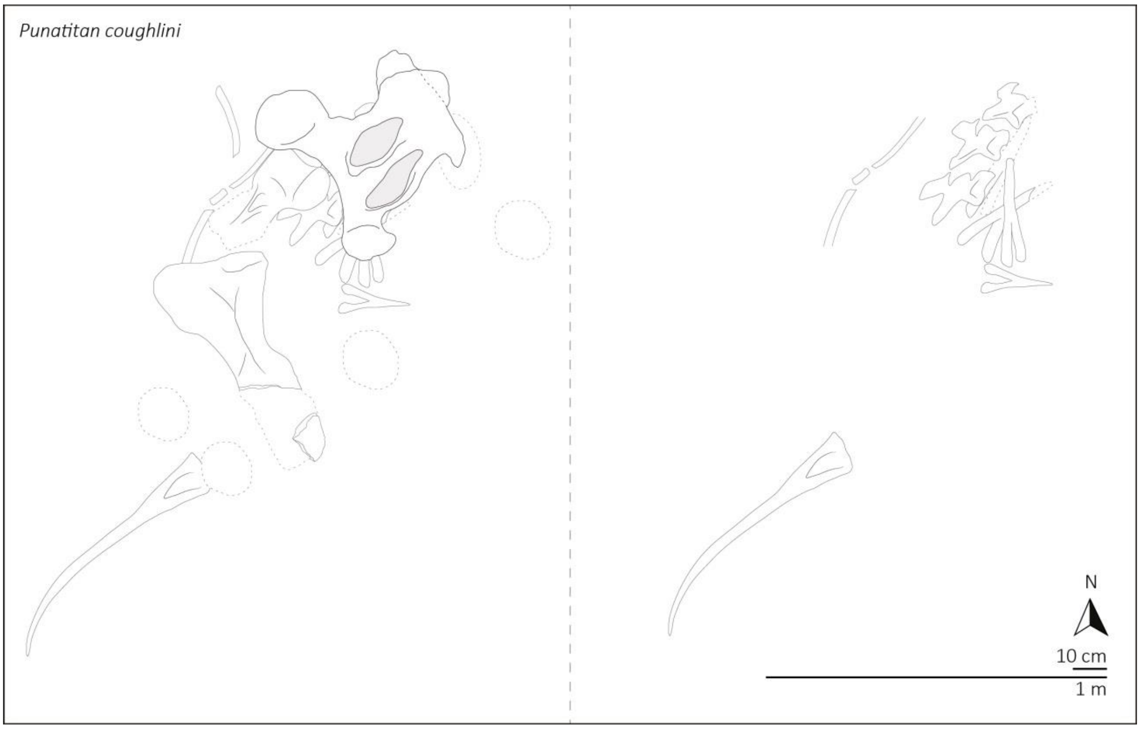

Supplementary Fig. 3. Outcrop map of the *Punatitan* gen. nov. holotype quarry. Distances between skeletal elements and their orientations are shown as observed in the field (not restored to horizontal). Dotted lines represent undetermined bone fragments.

Measurements

Measurements made on the holotype specimens of *Punatitan* and *Bravasaurus* are listed in Supplementary Table 1 and Supplementary Table 2, respectively.

Supplementary Table 1. Representative elements of *Punatitan* gen. nov. Measurements are in cm. Asterisks indicate dubious data because the exact limits of one or both landmarks are difficult to recognise.

Cervical vertebra

CRILAR-Pv 614/	*1*
Centrum height	8.5
Centrum width	21.5
Distance between prezygapophyses	6.5

Dorsal vertebrae

CRILAR-Pv 614/	*2*	*3*
Centrum length	20.7	-
Height of the posterior articular surface	16.5	-
Width of the posterior articular surface	15.4	-
Total height	45	-
Height of the neural spine (above the medial border of the postzygapophyses)	17.5	-

Caudal vertebrae

CRILAR-Pv 614/	*4*	*5*	*6*	*7*	*8*	*9*	*10*
Centrum length (excluding posterior articular surface)	-	9	8	8	8	8.5	8.5
Height of the posterior articular surface	13	12	11	8.5	7.5	7.5	7.5
Total height	-	-	23.5	24.5	22.5	18	16.5
Height of the neural spine (above the medial border of the postzygapophyses)	-	-	9	12.5	12	10	7

CRILAR-Pv 614/	*11*	*12*	*13*	*14*	*15*	*16*
Centrum length (excluding posterior articular surface)	9	8.5	8.5	8	8	-
Height of the posterior articular surface	7	7	6.5	6.5	6	-
Total height	13.5	13*	13.5	13.5*	-	-
Height of the neural spine (above the medial border of the postzygapophyses)	6.5	5.5*	-	-	-	-

Supplementary Table 2. Representative elements of *Bravasaurus* gen. nov. Measurements are in cm. Asterisks indicate dubious data because the exact limits of one or both landmarks are difficult to recognise.

Cervical vertebrae

CRILAR-Pv 612/	*1*	*2*	*3*	*4*
Centrum length	15.5	16.5	19	21
Centrum height	3.5	4.5	4.7	5.5
Centrum width	4	4.5	4.7	5.5
Total height	10	?	12.5	13.5
Distance between prezygapophyses	5	?	?	?

Dorsal vertebrae

CRILAR-Pv 612/	*5*	*6*	*7*	*8*	*9*
Centrum length	-	13.5*	-	-	-
Height of the posterior articular surface	7.5	7.5	8.5	10.7	9
Width of the posterior articular surface	9.5	10	13	11.5	10.7
Total height	20.5*	22.5	24.5*	26.5	25.5
Height of the neural spine (above the medial border of the postzygapophyses)	6.5*	8.7	12*	11.5	11.5

Caudal vertebrae

CRILAR-Pv 612/	*10*	*11*	*12*
Centrum length	9	8.3	8
Height of the posterior articular surface	5*	-	3.5
Width of the posterior articular surface	7*	5	4
Total height	12.5*	-	7.5
Height of the neural spine (above the medial border of the postzygapophyses)	3.3*	-	2.2

Forelimb

Humerus CRILAR-Pv 612/	*13*
Length	53
Maximum proximal width	24
Maximum distal width	18.5
Minimum section perimeter	24.5

Metacarpal CRILAR-Pv 612/	*14*
Length	19

Maximum proximal width	8
Maximum distal width	5.2

Pelvic girdle

Pubis CRILAR-Pv 612/	*15*
Length	47.5

Hind limb

Femur CRILAR-Pv 612/	*16*
Length	68
Maximum proximal width	23
Maximum distal width	22*
Minimum section perimeter	31

Fibulae CRILAR-Pv 612	*17*	*18*
Length	-	48.7
Maximum proximal width	-	10
Maximum distal width	8.2	8.3
Minimum section perimeter	11.5	12

Titanosauria in South America

In the Cretaceous Period, the titanosaurian clade reached a worldwide distribution (Cerda et al., 2012; Faria et al., 2015; Gorscak and O‘Connor, 2016). Particularly in South America, there are 51 valid species, of which nearly 75% were found in the Argentinean Patagonia (Supplementary Table 3).

Supplementary Table 3. Valid species of Cretaceous Titanosauria in South America, by regions.

NW Argentina + Chile	*Saltasaurus loricatus*
	Atacamatitan chilensis
	Punatitan coughlini gen. et sp. nov.
	Bravasaurus arrierosorum gen. et sp. nov.
Brazil	*Maxakalisaurus topai*
	'Aeolosaurus' maximus
	Uberabatitan ribeiroi
	Austroposeidon magnificus
	Trigonosaurus pricei
	Tapuiasaurus macedoi
	Baurutitan britoi
	Gondwanatitan faustoi
	Brasilotitan nemophagus
	Adamantisaurus mezzalirai
Patagonia	*Puertasaurus reuili*
	Argyrosaurus superbus
	Aeolosaurus colhuehuapensis
	Epachthosaurus sciuttoi
	Drusilasaura deseadensis
	Dreadnoughtus schrani
	Elaltitan lilloi
	Sarmientosaurus musacchioi
	Nullotitan glaciaris
	Antarctosaurus wichmannianus
	Andesaurus delgadoi
	Aeolosaurus rionegrinus
	Argentinosaurus huinculensis
	Barrosasaurus casamiquelai
	Bonitasaura salgadoi
	Bonatitan reigi
	Futalognkosaurus dukei
	Malarguesaurus florenciae
	Mendozasaurus neguyelap
	Quetecsaurus rusconii

	Notocolossus gonzalezparejasi
	Muyelensaurus pecheni
	Rinconsaurus caudamirus
	Narambuenatitan palomoi
	Neuquensaurus australis
	Overosaurus paradasorum
	Panamericansaurus schroederi
	Pellegrinisaurus powelli
	Petrobrasaurus puestohernandezi
	Pitekunsaurus macayai
	Rocasaurus muniozi
	Traukutitan eocaudata
	Baalsaurus mansillai
	Kaijutitan maui
	Choconsaurus baileywillisi
	Laplatasaurus araukanicus
Ecuador	*Yamanasaurus lojaensis*

Anatomical characters

Taxa added to the analysis. We added six taxa to the dataset provided by Carballido et al. (2020), including *Rocasaurus* (Salgado and Azpilicueta, 2000), *Aeolosaurus rionegrinus* (Powell, 2003), *Gondwanatitan* (Kellner and de Azevedo, 1999), *Uberabatitan* (Salgado and Carvalho, 2008; Silva et al., 2019), and *Punatitan* and *Bravasaurus* . Personal observations were also made on all these taxa.

Characters. We added just five characters to the original data matrix of 417 characters provided by Carballido et al. (2020). Two of them are from Salgado et al. (1997), one from Salgado et al. (2014), one from Santucci and Arruda-Campos (2011), and one is new. In addition, a few characters were slightly modified (Supplementary Table 4). Some scorings were also changed according to new observations and published data (Supplementary Data 1). We introduced 169 modifications to the dataset provided by Carballido et al. (2020), 80% of which were previously missing data. From 33 changes to the original scorings, 17 are due to modifications in the characters' definitions or their states (e.g., character 300). Among the other 16, three are changes to ambiguous states (e.g., *Overosaurus*, character 178), one to "not-applicable" and another to missing data. The remaining 11 modifications are listed in Supplementary Table 5.

Supplementary Table 4. Characters added or modified.

**Character**	**Modifications**
Ch. 141	One state added (state 2; see below).
Ch. 177	Modified definition (now it also considers the middle dorsal vertebrae).
Ch. 233	One state added (state 2; see below).
Ch. 250	One state added (state 3; see below)
Ch. 251	One state added (state 2; see below).
Ch. 254	One state added (state 2; see below).
Ch. 300	One state added (split state 1 into states 1 and 2; see below)
Ch. 418	Character added from Salgado et al., 1997.
Ch. 419	Character added from Salgado et al., 1997.
Ch. 420	Character added from Salgado et al., 2014.
Ch. 421	New Character.
Ch. 422	New Character.

Supplementary Table 5. Specific modifications.

Epachthosaurus	164	0→1	*Epachthosaurus* has prespinal laminae in its dorsal vertebrae.
Overosaurus	122	1→2	Modified based on Coria et al. (2013).
	223	1→0	Modified based on Coria et al. (2013). The transverse processes can be traced until caudal 16 o 17.
Muyelensaurus	159	1→3	Modified based on Calvo et al., 2007a
	175	0→1	The preserved anterior dorsal is not complete so that interpretations could be controversial (Calvo et al., 2007a). We interpret its condition as state 1.
Rinconsaurus	126	2→3	Modified based on Calvo and González Riga 2003. No evident pleurocoel is observed, but there is a deep fossa as observed in related forms.
Trigonosaurus	128	1→0	Epipophyses are not well developed as in saltasaurids (Campos et al., 2005).
	175	0→1	Modified after personal photos/observations of the specimens. Also, see Campos et al. (2005).
	196	1→0	Dorsal vertebrae 6-10 do not show aliform processes. A subtle expansion on one side of the neural spine in D5 might look like an aliform process, but it does not seem entirely consistent.
Baurutitan	237	2→1	The neural spine is slightly wider than long, but not as much as 1.5 times (Kellner et al., 2005).
	257	2→1	The neural spines are vertical (Kellner et al., 2005).

Phylogenetic analysis

We performed a maximum parsimony analysis of a modified version of the dataset of Carballido et al. (2020; see below) using TNT v. 1.1 (Goloboff et al., 2008). After doing a heuristic search with 1,000 replicates of Wagner trees and the first round of tree bisection-reconnection (TBR) branch swapping, we obtained 100 most parsimonious trees (MPTs). With another round of TBR the number of MPTs increased up to 150,000, once the memory overflowed. A strict consensus tree of 1480 steps (consistency index, 0.353; retention index, 0.724) is shown in Supplementary Fig. 4.

The present phylogenetic analysis recovers almost the same topology as previous studies (Canudo et al., 2018; Carballido et al., 2020, 2017; Supplementary Fig. 4). However, some inconsistencies about Late Cretaceous sauropods deserve a more detailed discussion. The main variations correspond to the affinities of *Andesaurus* with probably more basal taxa, the more basal position of *Malawisaurus*, outside Eutitanosauria, the colossosaurian affinities of *Baurutitan*, and the inclusion of *Gondwanatitan*, *Aeolosaurus rionegrinus*, *Uberabatitan*, *Trigonosaurus*, *Punatitan* and *Bravasaurus* as members of Rinconsauria and Aeolosaurini.

In the present analysis, *Andesaurus* is recovered as part of a large polytomy that includes brachiosaurids, euhelopodids and sister taxa of Lithostrotia, among others. A discrepancy regarding the position of *Andesaurus* could be linked to the presence of some taxa that have been previously recognized as unstable, such as *Padillasaurus*, *Malarguesaurus*, *Lusotitan* or *Rayososaurus* (Carballido et al., 2020, 2017). However, its affinities with other somphospondylans are beyond the scope of this paper.

Malawisaurus is recovered from the analysis as more basal than *Epachthosaurus* and, thus, outside Eutitanosauria. Such position contrasts with that obtained by Carballido et al. (2017) and Canudo et al. (2018). However, it is consistent with other analyses based on different data sets (González Riga et al., 2018; Salgado et al., 2014; Tykoski and Fiorillo, 2016).

The Brazilian titanosaurian *Baurutitan* has been included in a few phylogenetic analyses. Martínez et al. (2016) recovered its position within a major polytomy that consists of the saltasaurines *Saltasaurus*, *Rocasaurus* and *Neuquensaurus*, some basal titanosaurs, such as *Epachthosaurus*, and few other Brazilian forms, such as

Tapuiasaurus, *Gondwanatitan* and *Trigonosaurus*, among others. Based on a modified version of the latter data set, Silva et al. (2019) recognised *Baurutitan* as a non-saltasaurine titanosaurian, more closely related to *Rapetosaurus*. It is worth mentioning that their analysis only includes one member of Colossosauria: *Bonitasaura*. The study conducted by Bandeira et al. (2016) posits *Baurutitan* outside Titanosauria, although this taxon has evident titanosaurian affinities, such as the presence of strongly procoelous caudal centra (a synapomorphy of Titanosauria). Using a different data matrix, Carballido et al. (2017), and subsequent analyses derived from it (Canudo et al., 2018; Carballido et al., 2020), recovered *Baurutitan* as the sister taxon of a major polytomy that includes South American saltasaurines and related forms (*e.g. Opisthocoelicaudia*, *Nemegtosaurus*, *Alamosaurus*) plus *Rapetosaurus* + *Isisaurus* + *Tapuiasaurus*. The polytomy is apparently resolved by pruning *Nemegtosaurus* (Carballido et al., 2017), although, here, the removal of this taxon does not resolve the polytomy (see below; Supplementary Fig. 4). *Baurutitan* is here recovered as the most basal member of Colossosauria.

Despite the incorporation of *Rocasaurus* into the analysis, the relations of the saltasaurine titanosaurians remain obscure. In our phylogenetic result, they are recovered separately from a clade formed by *Rapetosaurus* (Madagascar), *Isisaurus* (India) and *Tapuiasaurus* (South America). As stated above, the same topology was obtained in a previous study after pruning *Nemegtosaurus* from the strict consensus tree (Carballido et al., 2017).

The main change compared to previous studies is the grouping of several Late Cretaceous titanosaurians from Bauru, plus *Bravasaurus* and *Punatitan*, within Rinconsauria. The clade that includes *Bonitasaura* + (*Notocolossus* + Lognkosauria) remains invariable with respect to the results obtained by Carballido et al. (2020, 2017). In contrast, the clade Rinconsauria has undergone several modifications. This clade originally included *Rinconsaurus* and *Muyelensaurus* (Calvo et al., 2007a; Salgado et al., 2014). It was further supported by Tykoski and Fiorillo (2016) and González Riga et al. (2018), who also recognised similarities with *Aeolosaurus rionegrinus* and *Gondwanatitan*. However, the latter taxa were not included in the data matrix provided by Carballido et al. (2020, 2017). Instead, their analyses recover the Patagonian *Overosaurus* and the Brazilian *'Aeolosaurus'* as members of Rinconsauria. Although the present data provide strong information about the invalidity of the Brazilian

‘*Aeolosaurus*’ genus, it can still be considered an Aeolosaurini (Martinelli et al., 2011). The results of the present study group *Rinconsaurus* and *Muyelensaurus* as successive, sister taxa of the clade Aeolosaurini. This clade is divided into two smaller clades. The first one includes *Overosaurus* + (‘*Aeolosaurus*’ + (*Aeolosaurus* + *Punatitan*)), whereas the other corresponds to an unresolved polytomy between *Gondwanatitan*, *Trigonosaurus*, *Uberabatitan* and *Bravasaurus*.

The incorporation of *Trigonosaurus* within Rinconsauria and the position of *Bravasaurus* and *Punatitan* within two independent but closely related clades are worth mentioning. As well as *Uberabatitan*, *Trigonosaurus* has been included in a few data matrices (Bandeira et al., 2016; Carballido et al., 2017; Silva et al., 2019). Bandeira et al. (2016) did not find direct affinities between *Trigonosaurus* and other Brazilian taxa, such as *Uberabatitan* or ‘*Aeolosaurus*’ *maximus*. Based on a data matrix modified from Martínez et al. (2016) Silva et al. (2019) recovered *Trigonosaurus* as a non-saltasaurine titanosaur, related with the saltasaurines plus *Epachthosaurus* and *Bonitasaura*. Using a different data set, Carballido et al. (2020, 2017) excluded *Trigonosaurus* from their reduced consensus tree after finding it unstable. Here, *Bravasaurus* and *Punatitan* (from La Rioja), *Overosaurus* and *Aeolosaurus* (from Patagonia), as well as *Trigonosaurus*, *Gondwanatitan*, ‘*Aeolosaurus*’, and *Uberabatitan* (from SW Brazil) are recovered as members of Aeolosaurini (Franco-Rosas et al., 2004). *Bravasaurus* shares several features with *Uberabatitan* (Salgado and Carvalho, 2008; Silva et al., 2019). The cervical, dorsal and caudal vertebrae of both taxa also show many similarities with those of *Trigonosaurus*, which is reflected in their position (although unresolved) in the strict consensus tree (Supplementary Fig. 4). *Punatitan* exhibits most of the typical features that allow recognition of aeolosaurine titanosaurians, e.g., caudal vertebrae with anteriorly oriented neural arches. In fact, the current analysis suggests that *Punatitan* could have been more related to *Aeolosaurus rionegrinus* than the Brazilian ‘*Aeolosaurus*’. On the other hand, although *Bravasaurus*, *Uberabatitan* and *Trigonosaurus* do not have typically aeolosaurine caudals, they are recovered in a polytomy with *Gondwanatitan* and, thus, they are also members of the clade Aeolosaurini.

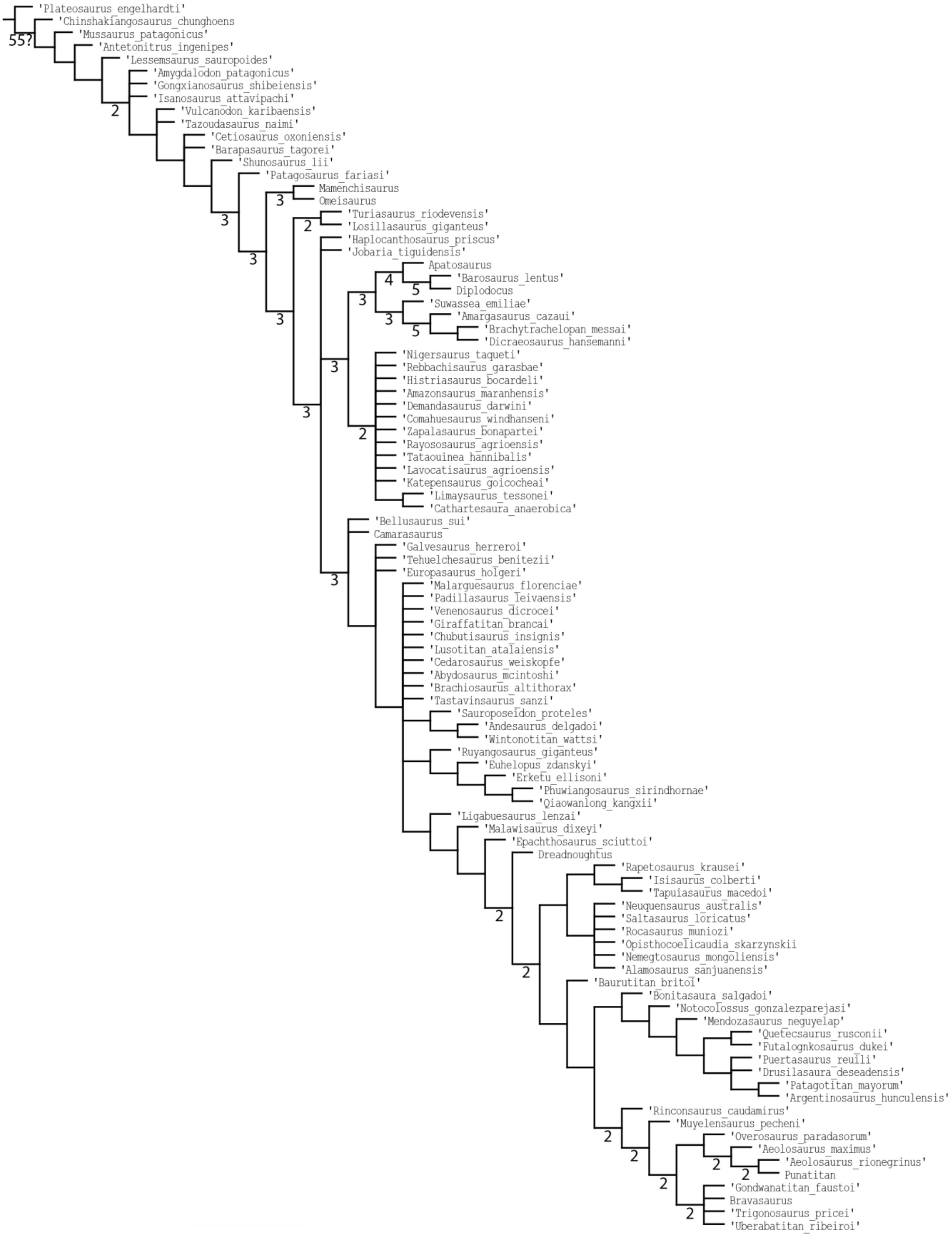

Supplementary Fig. 4. Strict consensus tree. From 150,000 most parsimonious trees, of 1480 steps. Bremer supports higher than one are shown on their respective branches.

Latitudinal position of South American taxa

Our phylogenetic analysis recovers 30 derived titanosaurians within Lithostrotia; 25 of them were found in different Cretaceous basins across South America. The latitudinal gradient is one of the most obvious patterns in biogeography (e.g., Pianka, 1966; Sax, 2001). We calculated the palaeogeographic coordinates for each South American taxon using the software GPlates (Müller et al., 2018; Seton et al., 2012) (Supplementary Table 6). We translated this data into a colour gradient, which we used in the phylogenetic tree in Fig. 4. The colour coding enhances the visualization of latitudinal differences between clades of derived titanosaurians. Then it is easy to observe, for example, that Lognkosauria is a strictly Patagonian clade. In contrast, the small clade of aeolosaurines that contains *Bravasaurus* seems to be restricted to lower latitudes, whereas the clade in which *Punatitan* is nested comprises a much larger latitudinal interval, between Patagonia and SW Brazil.

Supplementary Table 6. Latitude, palaeolatitude, location, and age of South American Lithostrotia included in the phylogenetic analysis.

Taxon	Latitude (deg.)	Palaeo lat. (deg.)	Basin	Max. age (My)	Min. age (My)	Reference
Tapuiasaurus	-16.7	-21.1	Sanfranciscana	125.0	113.0	Zaher et al., 2011
Uberabatitan	-19.6	-22.8	Bauru	72.1	66.0	Salgado and Carvalho, 2008
Trigonosaurus	-19.7	-23.0	Bauru	72.1	66.0	(Campos et al., 2005)
Baurutitan	-19.7	-23.0	Bauru	72.1	66.0	Kellner et al., 2005
Gondwanatitan	-22.1	-25.4	Bauru	83.5	66.0	Kellner and de Azevedo, 1999
Aeolosaurus'	-23.0	-26.3	Bauru	83.5	66.0	Santucci and Arruda-Campos, 2011
Saltasaurus	-26.1	-28.8	Salta Group	70.6	66.0	Bonaparte and Powell, 1980
Punatitan	-28.6	-31.6	?	75.8	66.0	This work
Bravasaurus	-28.6	-31.6	?	75.8	66.0	This work
Quetecsaurus	-34.1	-38.5	Neuquén	93.5	89.3	González Riga and Ortiz David, 2014
Mendozasaurus	-37.1	-41.3	Neuquén	89.3	85.8	González Riga, 2003
Notocolossus	-37.1	-41.0	Neuquén	89.3	83.5	González Riga et al., 2016
Muyelensaurus	-37.4	-41.3	Neuquén	89.3	83.5	Calvo et al., 2007a
Rinconsaurus	-37.4	-41.2	Neuquén	86.3	83.6	Calvo and González Riga, 2003
Overosaurus	-37.6	-41.4	Neuquén	86.3	83.6	Coria et al., 2013
Rocasaurus	-39.3	-42.4	Neuquén	83.5	66.0	Salgado and Azpilicueta, 2000
Aeolosaurus	-38.8	-41.9	Neuquén	83.6	66.0	Powell, 2003
Bonitasaura	-39.6	-43.4	Neuquén	86.3	83.6	Apesteguía, 2004
Futalognkosaurus	-38.5	-42.9	Neuquén	93.5	85.8	Calvo et al., 2007b
Neuquensaurus	-39.0	-42.8	Neuquén	86.3	83.6	Powell, 1992

Argentinosaurus	-38.9	-43.5	Neuquén	99.6	89.8	Bonaparte and Coria, 1993
Patagotitan	-43.8	-48.8	Somuncurá-Cañadón Asfalto	105.3	99.6	Carballido et al., 2017
Epachthosaurus	-45.3	-49.8	Golfo San Jorge	99.6	89.3	Powell, 1990
Drusilasaura	-46.7	-51.2	Golfo San Jorge	99.6	89.3	Navarrete et al., 2011
Puertasaurus	-49.9	-52.9	Austral	83.6	66.0	Novas et al., 2005
Dreadnoughtus	-49.9	-52.9	Austral	83.6	66.0	Lacovara et al., 2014

Character list

Skull

1. Posterolateral processes of premaxilla and lateral processes of maxilla, shape: without midline contact (0); with midline contact forming marked narial depression, subnarial foramen not visible laterally (1). (Wilson, 2002: character 1).

2. Premaxillary anterior margin shape: without step (0); with marked step but short step (1); with marked and long step (2). (Modified from Wilson, 2002: character 2).

3. Premaxila, ascending process shape in lateral view: convex (0); concave, with a large dorsal projection (1); sub-rectilinear and directed posterodorsally (2). (Whitlock, 2011: character 3).

4. Premaxilla, external surface: without anteroventrally orientated vascular grooves originating from an opening in the maxillary contact (0); vascular grooves present (1). (Whitlock, 2011: character 2).

5. Premaxilla-maxilla suture, shape: planar (0); twisted along its length, giving the contact a sinuous appearance in lateral view (1). (D'Emic, 2012: character 2).

6. Premaxilla, small finger-like, vertically oriented premaxillary process near anteromedial corner of external naris: (0) absent; (1) present. (D'Emic, 2012: character 3).

7. Maxillary border of external naris, length: short, making up much less than onefourth narial perimeter (0); long, making up more than one-third narial perimeter (1). (Wilson, 2002: character 3).

8. Maxilla, foramen anterior to the preantorbital fenestra: absent (0); present (1). (Zaher et al., 2011).

9. Preanteorbital fenestra: absent (0); present, being wide and laterally opened (1). (Modified from Wilson, 2002: character 4).

10. Subnarial foramen and anterior maxillary foramen, position: well distanced from one another (0); separated by narrow bony isthmus (1). (Wilson, 2002: character 5).

11. Antorobital fenestra: much shorter than orbital maximum diameter, less than 85% of orbit (0); subequal to orbital maximum diameter, greater than 85% orbit (1). (Modified from Wilson, 2002: character 6 following to Whitlock, 2011: character 13).

12. Antorbital fenestra, shape of dorsal margin: straight or convex (0); concave (1). (Whitlock, 2011: character 14).

13. Antorbital fossa: present (0); absent (1). (Wilson, 2002: character 7).

14. External nares position: terminal (0); retracted to level of orbit (1); retracted to a position between orbits (2). (Wilson, 2002: character 8).

15. External nares, maximum diameter: shorter (0); or longer than orbital maximum diameter (1). (Wilson, 2002: character 9).

16. Orbital ventral margin, anteroposterior length: broad, with subcircular orbital margin (0); reduced, with acute orbital margin (1). (Wilson, 2002: character 10).

17. Lacrimal, anterior process: present (0); absent (1). (Wilson, 2002: character 11).

18. Lacrimal, anteriorly projecting vertical plate of bone: absent (0); present (1). (D'Emic, 2012: character 4).

19. Jugal contribution to the ventral border of the skull: present and long (0); absent or very reduced (1). (Carballido et al., 2012: character 16).

20. Quadratojugal-maxilla contact: absent or small (0); broad (1). (Whitlock, 2011: character 10).

21. Jugal-ectopterygoid contact: present (0); absent (1). (Wilson, 2002: character 12).

22. Jugal, contribution to antorbital fenestra: absent (0); present, but very reduced (1); present and large, bordering approximately one-third its perimeter (2). (Modified from Wilson, 2002: character 13).

23. Quadratojugal, position of anterior terminus: posterior to middle of orbit (0); anterior margin of orbit or beyond (1). (Whitlock, 2011: character 30).

24. Quadratojugal, anterior process length: short, anterior process shorter than dorsal process (0); long, anterior process more than twice as long as dorsal process (1). (Wilson, 2002: character 32).

25. Quadratojugal, angle between anterior and dorsal processes: less than or equal to 90°, so that the quadrate shaft is directed dorsally (0); greater than 90°, approaching 130°, so that the quadrate shaft slants posterodorsally (1). (Whitlock, 2011: character 31).

26. Ventral edge of anterior surface of the quadratojugal: straight, not expanded ventrally (0); slightly expanded ventrally, forming a small bulge, which height is less than twice the ramus height (1); well expanded ventrally, forming a notorious bulge, which height is twice or more the minimum height of the ramus (2). (Modified from Upchurch et al., 2004: character 26).

27. Squamosal contribution to the supratemporal fenestra: present, the squamosal is well visible in dorsal view (0); reduced or absent (1). (Curry-Rogers, 2005: character 37).

28. Squamosal-quadratojugal contact: present (0); absent (1). (Wilson, 2002: character 31).

29. Squamosal, posteroventral margin: smooth (0); "with prominent, ventrally directed "prong"" (1). (Whitlock, 2011: character 37).

30. Prefrontal posterior process size: small, not projecting far posterior of frontal-nasal suture (0); elongate, approaching parietal (1). (Wilson, 2002: character 14).

31. Prefrontal, posterior process shape: flat (0); hooked (1). (Wilson, 2002: character 15).

32. Prefrontal, anterior process: absent (0); present (1). (Curry-Rogers, 2005: character 30).

33. Prefrontal-frontal contact width: large, equal or longer that the anteroposterior length of the prefrontal (0); narrow, less than half the anteroposterior length of the prefrontal (1). (Zaher et al., 2011: character 239).

34. Postorbital, ventral process shape: transversely narrow (0); broader transversely than anteroposteriorly (1). (Wilson, 2002: character 16).

35. Postorbital, posterior process: present (0); absent (1). (Wilson, 2002: character 17).

36. Postorbital, posterior margin articulating with the squamosal: with tapering posterior process (0); with a deep posterior process (1). (Zaher et al., 2011: character 245).

37. Frontal contribution to supratemporal fossa: present (0); absent (1). (Wilson, 2002: character 18).

38. Frontals, midline contact (symphysis): sutured (0); or fused in adult individuals (1). (Wilson, 2002: character 19).

39. Frontal, anteroposterior length: approximately twice (0); or less than minimum transverse breadth (1). (Wilson, 2002: character 20).

40. Frontal-nasal suture, shape: flat or slightly bowed anteriorly (0); V-shaped, pointing posteriorly (1). (Whitlock, 2011: character 21).

41. Frontals, dorsal surface: without paired grooves facing anterodorsally (0); grooves present, extend on to nasal (1). (Whitlock, 2011: character 22).

42. Frontal, contribution to dorsal margin of orbit: contribution to dorsal margin of orbit: less than 1.5 times the contribution of prefrontal (0); at least 1.5 times the contribution of prefrontal (1). (Whitlock, 2011: character 23).

43. Parietal occipital process, dorsoventral height: short, less than the diameter of the foramen magnum (0); deep, nearly twice the diameter of the foramen magnum (1). (Wilson, 2002: character21).

44. Parietal, contribution to post-temporal fenestra: present (0); absent (1). (Wilson, 2002: character 22).

45. Parietal, distance separating supratemporal fenestrae: less than the long axis of supratemporal fenestra, 0.8 or less (0); almost the same as the long axis of supratemporal fenestra 0.8-1.2 (1); much larger than the long axis of supratemporal fenestra, more than 1.2 (2). (Modified from Wilson, 2002: character 24).

46. Postparietal foramen: absent (0); present (1). (Wilson, 2002: character 23).

47. Paroccipital process distal terminus: straight, slightly expanded surface (0); rounded, tongue-like process (1). (Whitlock, 2011: character 42).

48. Supratemporal fenestra: present (0); absent (1). (Wilson, 2002: character 25).

49. Supratemporal fenestra, long axis orientation: anteroposterior (0); transverse (1). (Wilson, 2002: character 26).

50. Supratemporal fenestra, maximum diameter: much longer than (0); or subequal to that of foramen magnum (1). (Wilson, 2002: character 27).

51. Supratemporal region, anteroposterior length: temporal bar longer (0); or shorter anteroposteriorly than transversely (1). (Wilson, 2002: character 28).

52. Supratemporal fossa, lateral exposure: not visible laterally, obscured by temporal bar (0); visible laterally, temporal bar shifted ventrally (1). (Wilson, 2002: character 29).

53. Supraoccipital, sagital nuchal crest: broad, weakly developed (0); narrow, sharp and distinct (1). (Whitlock, 2011: character 45).

54. Laterotemporal fenestra, anterior extension: posterior to orbit (0); ventral to orbit (1). (Wilson, 2002: character 30).

55. Quadrate fossa: absent (0); present (1). (Wilson, 2002: character 33).

56. Quadrate fossa, depth: shallow (0); deeply invaginated (1). (Wilson, 2002: character 34).

57. Quadrate fossa, orientation: posterior (0); posterolateral (1). (Wilson, 2002: character 35).

58. Quadrate, articular surface shape: quadrangular in ventral view, oriented transversely (0); roughly triangular in shape or thin, crescent-shaped surface with anteriorly directed medial process (1).(Modified, based on Mannion et al., 2012 from Whitlock, 2011: character 32)

59. Quadrate, articular surface shape: quadrangular in ventral view, oriented transversely or roughly triangular in shape (0); thin, crescent-shaped surface with anteriorly directed medial process (1). (Modified, based on Mannion et al., 2012 from Whitlock, 2011: character 32).

60. Palatobasal contact, shape: pterygoid with small facet (0); dorsomedially orientated hook (1); or rocker-like surface for basipterygoid articulation (2). (Wilson, 2002: character 36).

61. Pterygoid, transverse flange (i.e. ectopterygoid process) position: posterior of orbit (0); between orbit and antorbital fenestra (1); anterior to antorbital fenestra (2). (Wilson, 2002: character37).

62. Pterygoid, quadrate flange size: large, palatobasal and quadrate articulations well separated (0); small, palatobasal and quadrate articulations approach (1). (Wilson, 2002: character 38)

63. Pterygoid, palatine ramus shape: straight, at level of dorsal margin of quadrate ramus (0); stepped, raised above level of quadrate ramus (1). (Wilson, 2002: character39).

64. Pterygoid, sutural contact with ectopterygoid: broad, along the medial or lateral surface (0); narrow, restricted to the anterior tip of the ectopterygoid (1). (Zaher et al., 2011: character 240)

65. Palatine, lateral ramus shape: plate-shaped (long maxillary contact) (0); rod-shaped (narrow maxillary contact) (1). (Wilson, 2002: character 40).

66. Epipterygoid: present (0); absent (1). (Wilson, 2002: character 41).

67. Vomer, anterior articulation: maxilla (0); premaxilla (1). (Wilson, 2002: character 42).

68. Supraoccipital, height: twice subequal to (0); or less than height of foramen magnum (1). (Wilson, 2002: character 43).

69. Paroccipital process, ventral non-articular process: absent (0); present (1). (Wilson, 2002: character 44).

70. Crista prootica, size: rudimentary (0); expanded laterally into dorsolateral process (1). (Wilson, 2002: character 45).

71. Basipterygoid processes, length: short, approximately twice (0); or elongate, at least four times basal diameter (1). (Wilson, 2002: character 46).

72. Basipterygoid processes, angle of divergence: approximately 45° (0); less than 30° (1). (Wilson, 2002: character 47).

73. Basal tubera, anteroposterior depth: approximately half dorsoventral height (0); sheet-like, 20% dorsoventral height (1). (Wilson, 2002: character 48).

74. Basal tubera, breadth: much broader than (0); or narrower than occipital condyle (1). (Wilson, 2002: character 49).

75. Basal tubera: distinct from basipterygoid (0); reduced to slight swelling on ventral surface of basipterygoid (1). (Whitlock, 2011: character 53).

76. Basal tubera, shape of posterior face: convex (0); slightly concave (1). (Whitlock, 2011: character 54)

77. Basioccipital depression between foramen magnum and basal tubera: absent (0); present (1). (Wilson, 2002: character 50).

78. Basisphenoid/basipterygoid recess: present (0); absent (1). (Wilson, 2002: character 51).

79. Basisphenoid/quadrate contact: absent (0); present (1). (Wilson, 2002).

80. Basisphenoid, sagital ridge between basipterygoid processes: absent (0); present (1). (Zaher et al., 2011: character 242).

81. Basipterygoid processes, orientation: perpendicular to (0); or angled approximately 45° to skull roof (1). (Wilson, 2002: character 53).

82. Basipterygoid, area between the basipterygoid processes and parasphenoid rostrum: is a mildly concave subtriangular region (0); forms a deep slot-like cavity that passes posteriorly between the bases of the basipterygoid processes (1). (Mannion et al., 2012: character 48).

83. Occipital region of skull, shape: anteroposteriorly deep, paroccipital processes oriented posterolaterally (0); flat, paroccipital processes oriented transversely (1). (Wilson, 2002: character 54).

84. Occipital condyle, lateral surface of the basioccipital: flat or slightly convex (0); strongly concave (1). (Remes et al., 2009: character 50).

85. Dentary, depth of anterior end of ramus: slightly less than that of dentary at midlength (0); 150% minimum depth (1). (Wilson, 2002: character 55).

86. Dentary, anteroventral margin shape: gently rounded (0); sharply projecting triangular process (1). (Wilson, 2002: character 56).

87. Dentary symphysis, orientation: angled 15° or more anteriorly to (0); or perpendicular to axis of jaw ramus (1). (Wilson, 2002: character 57).

88. Dentary, cross-sectional shape of symphysis: oblong or rectangular (0); subtriangular, tapering sharply towards ventral extreme (1); subcircular (2). (Whitlock, 2011: character 60).

89. Dentary, tuberosity on labial surface near symphysis: absent (0); present (1). (Whitlock, 2011: character 57).

90. Dentary, posteroventral process shape: single (0); divided (1). (D'Emic, 2012: character 10).

91. Mandible, coronoid eminence: strongly expressed, clearly rising above plane of dentigerous portion (0); absent (1). (Whitlock, 2011: character 62).

92. External mandibular fenestra: present (0); absent (1). (Wilson, 2002: character 58).

93. Surangular depth: less than twice (0); or more than two and one-half times maximum depth of the angular (1). (Wilson, 2002: character 59).

94. Surangular ridge separating adductor and articular fossae: absent (0); present (1). (Wilson, 2002: character 60).

95. Adductor fossa, medial wall depth: shallow (0); deep, prearticular expanded dorsoventrally (1). (Wilson, 2002: character 61).

96. Splenial posterior process, position: overlapping angular (0); separating anterior portions of prearticular and angular (1). (Wilson, 2002: character 62).

97. Splenial posterodorsal process: present, approaching margin of adductor chamber (0); absent (1). (Wilson, 2002: character 63).

98. Coronoid, size: extending to dorsal margin of jaw (0); reduced, not extending dorsal to splenial (1); absent (2). (Wilson, 2002: character 64).

99. Tooth rows, shape of anterior portions: narrowly arched, anterior portion of tooth rows V-shaped (0); broadly arched, anterior portion of tooth rows U-shaped (1); rectangular, tooth bearing portion of jaw perpendicular to jaw rami (2). (Wilson, 2002: character 65).

100. Tooth rows, length: extending to orbit (0); restricted anterior to orbit (1); restricted anterior to antorbital fenestra (2); restricted anterior to subnarial foramen (3). (Modified from Wilson, 2002: character 66).

101. Maxillary teeth shape: straight along axis (0); twisted axially through an arc of 30-45°: absent (0); present (1). (D'Emic, 2012: character 15).

102. Dentary teeth, number: greater than 20 (0); 10-17 (1); 9 or fewer (2). (Modified from Wilson, 2002: character73).

103. Replacement teeth per alveolus, number: two or fewer (0); more than four (1). (Wilson, 2002: character 74).

104. Lateral plate: absent (0); present (1). (Upchurch et al., 2004: character 9).

105. Teeth, orientation: perpendicular (0); or oriented anteriorly relative to jaw margin (1). (Wilson, 2002: character 75).

106. Tooth crowns, orientation: aligned along jaw axis, crowns do not overlap (0); aligned slightly anterolingually, tooth crowns overlap (1). (Wilson, 2002: character 69).

107. Tooth crowns, shape: narrow crowns (0); broad crowns (1). (Carballido et al., 2017).

108. Tooth crowns, cross-sectional shape at mid-crown: elliptical (0); D-shaped (1); subcylindrical (2); cylindrical (3). (Wilson, 2002: character 70).

109. SI values for tooth crowns: less than 3.0 (0); 3.0-4.0 (1); 4.0-5.0 (2); more than 5.0 (3). (Upchurch et al., 2004: characters 67-69).

110. Crown-to-crown occlusion: absent (0); present (1). (Wilson, 2002: character 67).

111. V-shaped wear facets: present (0); absent (1). (Modified from Wilson, 2002: character 68).

112. Development of the marginal wear facets: well developed (0); slightly developed as marginal facets (1).(Carballido et al., 2017)

113. One high angle wear facet and a second low angle wear facet: absent (0); present (1). (Carballido et al., 2017)

114. Single planar wear facet in labial or lingual surface of the teeth: absent (0); present (1). (Carballido et al., 2017)

115. Marginal tooth denticles: present (0); absent on posterior edge (1); absent on both anterior and posterior edges (2). (Wilson, 2002: character 72).

116. Enamel surface texture: smooth (0); wrinkled (1). (Wilson, 2002: character71).

117. Thickness of enamel asymmetric labiolingually: absent (0); present (1). (Whitlock, 2011: character 74).

118. Teeth, longitudinal grooves on lingual aspect: absent (0); present (1). (Wilson, 2002: character 76).

Cervical vertebrae

119. Cervical vertebrae, number: 10 or fewer (0); 12 (1); 13-14 (2); 15 (3); 16 or more (4). (Modified from Wilson, 2002: character 80 and Upchurch et al., 2004: characters 96-100).

120. Atlas, intercentrum occipital facet shape: rectangular in lateral view, length of dorsal aspect subequal to that of ventral aspect (0); expanded anteroventrally in lateral view, anteroposterior length of dorsal aspect shorter than that of ventral aspect (1). (Wilson, 2002: character 79).

121. Axis, centrum shape: over two and a half times as long as tall (0); less than twice as long as tall (1). (D’Emic, 2012: character 20).

122. Cervical vertebrae, parapophyses, shape and orientation: short and weakly developed, projected laterally or slightly ventrally (0); middle development, ventrally such that the cervical ribs are displaced ventrally around half the height of the centrum (1); well developed, broad and ventrally projected such that cervical ribs are displaced ventrally more than the height of the centrum (2). (Modified from D’Emic, 2012: character 29).

123. Cervical centra, articulations: amphicoelous (0); opisthocoelous (1). (Salgado et al., 1997: character 1; Wilson, 2002: character 82; Upchurch, 1998: character 81 and Upchurch et al., 2004: character 103).

124. Cervical centra, ventral surface: is flat or slightly convex transversely (0); transversely concave (1). (Upchurch, 1998: character 84 and Upchurch et al., 2004: character 107).

125. Cervical centra, midline keels on ventral surface: prominent and plate-like (0); reduced to low ridges or absent (1). (Upchurch, 1998: character 83 and Upchurch et al., 2004: character 106).

126. Cervical centra, pleurocoels: absent (0); present with well-defined anterior, dorsal, and ventral edges, but not the posterior one (1); present, with well-defined edges (2); absent, but with deep lateral fossa which bears small pneumatopores that communicate to the interior pneumatic cavities (3). (Carballido et al., 2012)

127. Cervical centra, pleurocoels: singles without division (0); with a well-defined anterior excavation and a posterior smooth fossa (1); divided by a bone septum, resulting in an anterior and a posterior lateral excavation (2); divided in three or more lateral excavations, resulting in a complex morphology (3); with a well-defined anterior excavation and a posterior smooth fossa (Harris, 2006; Modified from Salgado et al., 1997; Wilson, 2002).

128. Cervical vertebrae, well developed epipophyses: absent (0); present (1). (Carballido et al., 2017)

129. Cervical vertebrae, epipophyses shape: stout, pillar-like expansions above postzygapophyses (0); posteriorly projecting prongs (1). (D’Emic, 2012: character 24).

130. Prezygapophyses, anterior process situated ventrolaterally to the articular surface: absent (0); present (1). (Remes et al., 2009: character 79).

131. Cervical vertebrae with an accessory lamina, which runs from the PODL (or slightly anteriorly) up to the SPOL: absent (0); present (1). (Modified from D'Emic, 2012: character 25).

132. Cervical vertebrae, height divided width (measured in its posterior articular surface): higher than 1.1 (0), around 1 (1); between 0.9 and 0.7 (2); smaller than 0.7 (3). (Modified from Wilson, 2002: character 84; Upchurch, 1998: character 85 and Upchurch et al., 2004: character 108).

133. Cervical centra, small notch in the dorsal margin of the posterior articular surface: absent (0); present (1). (Carballido et al., 2012).

134. Cervical vertebrae, neural arch lamination: well developed, with well-marked laminae and fossae (0); rudimentary, with diapophyseal laminae absents or very slightly marked (1). (Wilson, 2002: character 81).

135. Cervical vertebrae with an accessory lamina, which runs from the postzygodiapophyseal lamina (PODL) up to the spinoprezygapophyseal lamina (SPRL): absent (0); present (1). (Modified from Sereno et al., 2007: characters 50, 51; Whitlock, 2011: characters 78, 96).

136. Cervical centra, internal pneumaticity: absent (0); present with singles and wide cavities (1); present, with several small and complex internal cavities (2). (Modified from Carballido et al., 2011).

137. Anterior cervical vertebrae, prespinal lamina: absent (0); present (1). (Carballido et al., 2012).

138. Anterior cervical vertebrae, neural spine shape: single (0); bifid (1). (Wilson, 2002: character 72; Upchurch et al., 2004: character 118).

139. Middle and posterior cervical vertebrae, prespinal lamina: absent (0); present (1). (Carballido et al., 2012).

140. Middle cervical vertebrae, lateral fossae on the prezygapophysis process: absent (0); present (1). (Harris, 2006).

141. Middle, cervical vertebrae, height of the neural arch: less than the height of the posterior articular surface (0); higher than the height of the posterior articular surface, with spine lower than distance between postzygapophysis and the dorsal margin of the centrum (1); higher, with spine lower than distance between postzygapophysis and the dorsal margin of the centrum (2). (Wilson, 2002: character 87; similar Upchurch et al., 2004: 111 and 112). Modified here. In *Bravasaurus* and *Trigonosaurus*, among other derived titanosaurians, the neural arch is taller than the posterior articular surface, but the segment above the postzygapophyses is relatively low compared to other titanosaurians.

142. Middle cervical centrum, anteroposterior length divided the height of the posterior articular surface: less than 4 (0); more than 4 (1). (Wilson, 2002: character 74; and Upchurch et al., 2004: character 102).

143. Middle and posterior cervical vertebrae, morphology of the centroprezygapophyseal lamina: single (0); dorsally divided, resulting in a lateral and medial lamina, being the medial lamina linked with the intraprezygapophyseal lamina and not with the prezygapophysis (1); divided, resulting in the presence of a "true" divided centroprezygapophyseal lamina, which is dorsally connected to the prezygapophysis (2). (Carballido et al., 2012).

144. Middle and posterior cervical vertebrae, morphology of the centropostzygapophyseal lamina (CPOL): single (0); divided, with the medial part contacting the intrapostzygapophyseal lamina (1) (Carballido et al., 2012).

145. Middle and posterior cervical vertebrae, articular surface of zygapophyses: flat (0); transversally convex (1). (Upchurch et al., 2004).

146. Middle and posterior cervical vertebrae, prominent triangular flange on posterior edge of the diapophyseal process (in the PCDL): absent (0); present (1). (Remes et al., 2009: character 78).

147. Middle cervical vertebrae, prezygapophyses position: do not extend beyond the anterior margin of the centrum (0); extends beyond the anterior margin of the centrum (1). (Salgado et al., 1997, character 37).

148. Middle and posterior cervical vertebrae, parapophysis shape: subcircular (0); anteroposteriorly elongate (1). (D'Emic, 2012: character 28).

149. Posterior cervical vertebrae, lateral profile of the neural spine: displays steeply sloping cranial and caudal faces (0); displays steeply sloping cranial face and noticeably less steep caudal margin (1). (Upchurch et al., 2004: character 119).

150. Posterior cervical vertebrae, neural spine shape: not expanded distally (0); expanded but not as much as the width of the centrum (1); laterally expanded, being equal or wider than the vertebral centrum (1). (Modified from González Riga et al., 2009).

151. Posterior cervical vertebrae, lateral expansion: SPRLs does not contact the lateral margins of the neural spine (0); SPRLs are contacting the lateral margins of the neural spine (1). (Modified from González Riga and Ortiz David, 2014: characters 26-27).

152. Posterior cervical and anterior dorsal vertebrae, neural spine shape: single (0); bifid (1). (Wilson, 2002: character 90, Upchurch et al., 2004: character 118).

153. Posterior cervical vertebrae, proportions – ratio total height /centrum length: less than 1.5 (0); more than 1.5 (1). (González Riga et al., 2009: character 32).

154. Posterior cervical and anterior dorsal bifid neural spines, median tubercle: absent (0); present (1). (Carballido et al., 2012: character 133)

Dorsal vertebrae

155. Number of dorsal vertebrae: 14 or more (0); 13 (1); 12 (2); 10 (3). (Modified from Wilson, 2002: character 91; Upchurch et al., 2004: characters 122- 125).

156. Dorsal centra, pleurocoels: absent (0); present (1). (Wilson, 2002: character 78; Upchurch et al., 2004: character 128).

157. Dorsal vertebrae, transverse processes: are directed laterally or slightly upwards (0); are directed strongly dorsolaterally (1). (Upchurch et al., 2004: character 138).

158. Dorsal vertebrae, distal end of the transverse process: curves smoothly into the dorsal surface of the process (0); is set off from the dorsal surface, the latter having a distinct dorsally facing flattened area (1). (Upchurch et al., 2004: character 140).

159. Anterior dorsal vertebrae, non-bifid neural spine in anterior or posterior view: possess subparallel lateral margins (0); possess lateral margins which slightly diverge dorsally (1); possess lateral margins which strongly diverge dorsally (2). (Modified 52 from Wilson, 2002: character 107; Upchurch et al., 2004: character 155).

160. Middle to posterior dorsal vertebrae, non-bifid neural spine in anterior or posterior view: possess subparallel lateral margins (0); possess lateral margins which slightly diverge dorsally (1); possess lateral margins which strongly diverge dorsally (2). (Modified from Wilson, 2002: character 107; Upchurch et al., 2004: character 155).

161. Dorsal centra, pneumatic structures: absent, dorsal centra with solid internal structure (0); present, dorsal centra with simple and big air-spaces (camerate) (1); present, dorsal centra with small and complex air-spaces (polycamerate) (2); present, dorsal centra with small and complex air spaces (semicamellate/camellate) (3). (Modified from Carballido et al., 2011).

162. Anterior and middle dorsal neural spines, spinoprezygapophyseal lamina (SPRL): absent (0); present (1). (Modified from Upchurch et al., 2007: character 131).

163. Posterior dorsal neural spines, spinoprezygapophyseal lamina (SPRL): absent (0); present (1). (Modified from Upchurch et al., 2007: character 132).

164. Dorsal vertebrae, single not bifid neural spines, single prespinal lamina (PRSL): absent (0); present (1). (Modified from Salgado et al., 1997: character14).

165. Dorsal vertebrae, single not bifid neural spines, single prespinal lamina (PRSL): rough and wide, present in the dorsalmost part of the neural spine (0); rough and wide, extended through almost all the neural spine (1); smooth and narrow (2). (Carballido et al., 2012).

166. Dorsal vertebrae with single neural spines, middle single fossa projected through the midline of the neural spine: present (0); absent (1). (Carballido et al., 2012).

167. Dorsal vertebrae with single neural spines, middle single fossa, projected through the midline of the neural spine: relatively wide median simple fossa (0); a thin median simple fossa (1); extremely reduced median simple fossa (2). (Carballido et al., 2012).

168. Anterior dorsal centra, articular face shape: amphicoelous (0); opisthocoelous (1). (Wilson, 2002: character 94; Upchurch et al., 2004: character 104).

169. Anterior and middle dorsal centra, pleurocoels: have rounded caudal margins (0); have tapering, acute caudal margins (1). (Salgado et al., 1997; Upchurch, 1998: character 06; Upchurch et al., 2004: character 127).

170. Middle dorsal neural arches in lateral view, anterior edge of the neural spine: project anteriorly to the diapophysis (0); converge with the diapophysis (1); project posteriorly to the diapophysis (2). (Carballido et al., 2012).

171. Anterior and middle dorsal vertebrae, zygapophyseal articulation angle: horizontal or slightly posteroventrally oriented (0); posteroventraly oriented (around 30º) (1); strongly posteroventraly oriented (more than 40º) (2). (Carballido et al., 2012).

172. Anterior dorsal vertebrae, neural spine orientation: vertical, or slightly inclined (less than 20º) (0); posterodorsally, more than 20º (1); anteriorly directed (2). (Wilson, 2002: character 102; Upchurch et al., 2004: characters 153-154)

173. Anterior dorsal vertebrae neural spine, triangular aliform processes: absent (0); present but do not project far laterally (not as far as caudal zygapophyses) (1); present and project far laterally (as far as caudal zygapophyses) (2). (Modified from Wilson, 2002: character 102 and Upchurch et al., 2004: characters 153-154).

174. Anterior dorsal vertebrae, neural spine minimums width / length: 0.5 or greater (stout and short neural spine) (0); lower than 0.5 (thin and tall neural spines). (Carballido et al., 2017: character 174).

175. Anterior dorsal vertebrae, neural spine length (from TPRL to top): less than the height of the centrum (0); slightly higher than the centrum (1); twice or more the height of the centrum (2). (Carballido et al., 2017: character 175).

176. Anterior dorsal vertebrae, dorsal edge of the neural spine: flat (0); arrow-shaped (1); convex (2). (Carballido et al., 2017: character 176).

177. Middle to posterior dorsal vertebrae, dorsal edge of the neural spine: flat (0); arrow-shaped (1); convex (2). (Modified from Carballido et al., 2017: character 177). Modified here to include middle dorsal vertebrae.

178. Middle to posterior dorsal centra, ventral surface: convex transversely (0); flattened (1); is slightly concave, sometimes with one or two crests (2). (Upchurch et al., 2004).

179. Middle dorsal vertebrae, hyposphene-hypantrum system: present (0); absent (1). (Modified from Salgado et al., 1997: character 25; Wilson, 2002: character 106; Upchurch et al., 2004: character 145).

180. Posterior dorsal vertebrae, hyposphene-hypantrum system: present and well developed, usually with a rhomboid shape (0); present and weakly developed, mainly as a laminar articulation (1); absent or only present in posteriormost dorsal vertebrae (2). (Carballido et al., 2012).

181. Middle and posterior dorsal vertebrae, transverse processes length: short (0); long (projecting along 1.5 the articular surface width) (1). (Carballido et al., 2012).

182. Mid and posterior dorsal vertebrae with a single lamina (the single TPOL) supporting the hyposphene or postzygapophysis from below: absent (0); present (1). (Modified from Upchurch et al., 2004: character 146).

183. Middle and posterior dorsal vertebrae, neural canal in anterior view: entirely surrounded by the neural arch (0); enclosed in a deep fossa, enclosed laterally by pedicels (1). (Upchurch et al., 2004: character 136).

184. Middle and posterior dorsal vertebrae, neural spine height: approximately twice the centrum length (0); for times the centrum length (1). (Upchurch et al., 2004).

185. Middle and posterior dorsal neural spines orientation: vertical (0); slightly inclined, with an angle of around 70 degrees (1); strongly inclined, with an angle not larger than 40 degrees (2). (Modified from Wilson, 2002: character 104).

186. Middle and posterior dorsal vertebrae, central keel: absent (0); present (1). (D'Emic, 2012: character 49).

187. Dorsal vertebrae, height of the neural arch divided the height of the centrum: less than 0.8 (0); more than 0.8 (1). (Pol et al., 2011).

188. Middle to posterior dorsal vertebrae, pleurocoel dorsal margin: rounded (0); angular (1). (Rauhut et al., 2015: character 346).

189. Middle to posterior dorsal vertebrae, pleurocoel dorsal margin: well below the dorsal margin of the centrum (0); at the level of the dorsal margin of the centrum or higher (1). (Rauhut et al., 2015: character 347).

190. Middle to posterior dorsal vertebrae, small fossa anterior or anteroventral to the pleurocoel: absent (0); present (1). (Rauhut et al., 2015: character 348).

191. Middle and posterior dorsal neural arches, centropostzygapophyseal lamina (CPOL), shape: simple (0); divided (1). (Wilson, 2002: character 95).

192. Middle and posterior dorsal neural arches, anterior centroparapophyseal lamina (ACPL): absent (0); present (1). (Wilson, 2002: character 96; Upchurch et al., 2004: character 133).

193. Middle and posterior dorsal neural arches, prezygoparapophyseal lamina (PRPL): absent (0); present (1). (Wilson, 2002: character 97).

194. Middle and posterior dorsal neural arches, posterior centroparapophyseal lamina (PCPL): absent (0); present (1). (Wilson, 2002: character 98, Upchurch et al., 2004: character 137).

195. Middle and posterior dorsal centrum in transverse section (height: width ratio): subcircular (ratio, similar to 1 or a bit higher) (0); slightly dorsoventrally compressed (ratios between 0.8 and 1) (1); strongly compressed (ratios below 0.8) (2). (Modified from Upchurch et al., 2004).

196. Middle and posterior dorsal vertebrae neural spine, triangular aliform processes: absent (0); present but do not project far laterally (not as far as caudal zygapophyses) (1); present and project far laterally (as far as caudal zygapophyses) (2). (Modified from Wilson, 2002: character 102 and Upchurch et al., 2004: characters 153-154).

197. Middle and posterior dorsal vertebrae, spinodiapophyseal lamina (SPDL): absent (0); present (1). (Upchurch et al., 2004: character 157).

198. Middle and posterior dorsal vertebrae, accessory spinodiapophyseal lamina (SPDL): absent (0); present (1). (Upchurch et al., 2004: character 151).

199. Dorsal vertebrae, spinodiapophyseal webbing: lamina follows curvature of neural spine in anterior view (0); lamina "festooned" from spine, dorsal margin does not closely follow shape of neural spine and diapophysis (1). (Whitlock, 2011: character 104).

200. Anterior dorsal vertebrae, spinopostzygapophyseal lamina (SPOL): absent (0); present (1). (Upchurch et al., 2007: character 133).

201. Middle and posterior dorsal neural spines, lateral spinopostzygapophyseal lamina (lSPOL): absent (0); present (1). (Wilson, 2002: 100; Upchurch et al., 2004: character 159).

202. Middle and posterior dorsal neural arches, spinodiapophyseal lamina (SPDL) and spinopostzygapophyseal lamina (lSPOL) contact: absent (0); present (1). (Wilson, 2002: character 101).

203. Middle and posterior dorsal vertebrae, spinodiapophyseal (SPDL) and spinopostzygapophyseal lamina (lSPOL) contact: ventral, well separated from the triangular aliform process (0); dorsal, forms part of the triangular aliform process (1). (Carballido et al., 2012).

204. Middle and posterior dorsal vertebrae, height of neural arch below the postzygapophyses (pedicel): less than height of centrum (0); subequal to or greater than height of centrum (1). (Whitlock, 2011: character 109).

205. Posterior dorsal vertebrae, medial spinopostzygapophyseal lamina (mSPOL): absent (0); present and forms part of the median posterior lamina (1). (Carballido et al., 2012).

206. Posterior dorsal vertebrae, transverse processes: lie posterior, or posterodorsal, to the parapophysis (0); lie vertically above the parapophysis (1). (Upchurch et al., 2004: character 139).

207. Posterior dorsal centra, articular face shape: amphicoelous (0); slightly opisthocoelous (1); opisthocoelous (2). (Modified from Wilson, 2002: character 105).

208. Posterior dorsal vertebrae, neural spine: narrower transversely than anteroposteriorly (0); broader transversely than anteroposteriorly (1). (Wilson, 2002: character 92).

209. Posterior dorsal vertebra, posterior centrodiapophyseal lamina (PCDL): has an unexpanded ventral tip (0); expands and may bifurcate toward its ventral tip (1). (Salgado et al., 1997).

Ribs

210. Cervical ribs, distal shafts of longest cervical ribs: are elongate and form overlapping bundles (0); are short and do not project beyond the caudal end of the centrum to which they are attached (1). (Wilson, 2002: character 140).

211. Cervical ribs, angle between the capitulum and tuberculum: greater than 90°, so that the rib shaft lies close to the ventral edge of the centrum (0); less than 90°, so that the rib shaft lies below the ventral margin of the centrum (1). (Wilson, 2002: character 139).

212. Dorsal ribs, proximal pneumatopores: absent (0); present (1). (Wilson, 2002: character 141)

213. Anterior dorsal ribs, cross-sectional shape: subcircular (0); plank-like, anteroposterior breadth more than three times mediolateral breadth (1). (Wilson, 2002).

Sacrum

214. Sacral vertebrae, number: 3 or fewer (0); 4 (1); 5 (2); 6 (3). (Wilson, 2002: character 108).

215. Sacrum, sacricostal yoke: absent (0); present (1). (Wilson, 2002: character 109).

216. Sacral vertebrae contributing to acetabulum: numbers 1-3 (0); numbers 2-4 (1). (Wilson, 2002: character 110).

217. Sacral neural spines length: approximately twice length of centrum (0); approximately four times length of centrum (1). (Wilson, 2002: character 111).

218. Sacral ribs, dorsoventral length: low, not projecting beyond dorsal margin of ilium (0); high extending beyond dorsal margin of ilium (1). (Wilson, 2002: character 112).

219. Pleurocoels in the lateral surfaces of sacral centra: absent (0); present (1). (Upchurch et al., 2004: character 165).

Caudal vertebrae

220. Caudal vertebrae, number: 35 or fewer (0); 40 to 55 (1); increased to 70-80 (2). (Wilson, 2002: character114).

221. Caudal bone texture: solid (0); spongy (camellate), with large internal cells (1). (Wilson, 2002: character 113).

222. Anterior caudals, pneumatized neural arch: absent (0); present (1).

223. Caudal transverse processes: persist through caudal 20 or more posteriorly (0); disappear by caudal 15 (1); disappear by caudal 10 (2). (Wilson, 2002: character 115).

224. First caudal centrum anterior articular surface: flat (0); concave (1); convex (2). (Carballido et al., 2017).

225. First caudal centrum, posterior articular surface: flat (0); concave (1); convex (2). (Carballido et al., 2017).

226. First caudal neural arch, coel on lateral aspect of neural spine: absent (0); present (1). (Wilson, 2002: character 117).

227. Anterior caudal vertebrae (mainly the first and second): ventral bulge on transverse process: absent (0); present (1). (D'Emic, 2012: character 52).

228. Anterior and middle caudal vertebrae, blind fossae in lateral centrum: absent (0); present (1). (D'Emic, 2012: character 56).

229. Posteriormost anteriors and middle caudal vertebrae, transverse processes orientation: perpendicular (0); swept backwards, reaching the posterior margin of the centrum (1). (D'Emic, 2012: character 59).

230. Anterior caudal vertebrae, transverse processes: ventral surface directed laterally or slightly ventrally (0); directed dorsally (1). (Whitlock, 2011: character 125).

231. Anterior caudal centra (excluding the first), articular face shape: amphiplatyan or amphicoelous (0); procoelous/distoplatyan (1); slightly procoelous (2); procoelous (3); posterior surface markedly more concave than the anterior one (4). (Modified from González Riga et al., 2009).

232. Anterior caudal centra, pleurocoels: absent (0); present (1). (Wilson, 2002: character 119).

233. Anterior caudal vertebrae, ventral surfaces: convex transversely (0); concave transversely (1); flat or slightly concave (2). (Modified from Upchurch et al., 2004: character 182). Modified so as to distinguish the concave surface of the caudal vertebrae of some titanosaurians like *Punatitan* and *Aeolosaurus* from others with flat or practically flat (but not convex) surfaces like *Uberabatitan* and *Baurutitan*.

234. Anterior and middle caudal vertebrae, ventrolateral ridges: absent (0); present (1). (Upchurch et al., 2004: character 183).

235. Anterior and middle caudal vertebrae, triangular lateral process on the neural spine: absent (0); present (1). (Whitlock, 2011: character 123).

236. Anterior caudal transverse processes shape: triangular, tapering distally (0); "winglike", not tapering distally (1). (Wilson, 2002: character 128).

237. Anterior caudal neural spines, transverse breadth: approximately 50% of (0); or greater than anteroposterior length (1). (Wilson, 2002: character 126).

238. Anterior caudal transverse processes, proximal depth: shallow, on centrum only (0); deep, extending from centrum to neural arch (1). (Wilson, 2002: character 127).

239. Anterior caudal transverse processes, diapophyseal laminae (ACDl, PCDL, PRDL, PODL): absent (0); present (1). (Wilson, 2002: character 129).

240. Anterior caudal transverse processes, anterior centrodiapophyseal lamina (ACDL), shape: single (0); divided (1). (Wilson, 2002: character 130).

241. Anterior caudal vertebrae, hyposphene ridge: absent (0); present (1). (Upchurch et al., 2004: character 187).

242. Anterior caudal centra, length: approximately the same (0); or doubling over the first 20 vertebrae (1). (Wilson, 2002: character 120).

243. Anterior caudal neural arches, spinoprezygapophyseal lamina (SPRL): absent, or present as small short ridges that rapidly fade out into the anterolateral margin of the spine (0); present, extending onto lateral aspect of neural spine (1); present, well developed and extending onto the anterior or anterolateral edges of the neural spine (2) (Modified from Wilson, 2002: character 121). A third state was incorporated in order to include the morphology observed in some taxa in which the SPRL is well developed, but is not extending into the lateral aspect of the neural spine, as is the case of *Patagotitan*.

244. Anterior caudal neural arches, spinodiapophyseal lamina (SPDL): absent (0); present (1). In titanosaurians the SPDL, when present, is extending from the

dyapophyseal section of the transverse process (the dorsalmost part of it) up to the neural spine. (Carballido et al., 2017).

245. Anterior caudal neural arches, spinoprezygapophyseal lamina (SPRL)-spinopostzygapophyseal lamina (SPOL) contact: absent (0); present, forming a prominent lamina on lateral aspect of neural spine (1). (Wilson, 2002: character 122).

246. Anterior caudal neural arches, prespinal lamina (PRSL): absent (0); present (1). (Wilson, 2002: character 123). (Carballido et al., 2017).

247. Anterior caudal vertebrae, ventral and medially placed SPRL, usually described as bifurcated PRSL: absent (0); present (1). (Carballido et al., 2017).

248. Anterior caudal prespinal lamina (PRSL), triangular shaped product of a dorsal expansion of it: absent (0); present (1). (Carballido et al., 2017).

249. Anterior caudal vertebrae, pair thin laminae that are bounding the prespinal laminae and that diverge dorsally: absent (0); present (1). (Carballido et al., 2017).

250. Middle caudal centra, shape: cylindrical (0); with flat ventral margin (1); quadrangular, flat ventrally and laterally (2); trapezoidal (laterally compressed forming a shallow fossa) (3). (Modified from Wilson, 2002: character 131 and Carballido et al., 2020: character 250). We added a state that differentiates a centrum with a square profile, as in some more primitive sauropods (e.g. *Diplodocus*), from the centrum with a trapezoidal profile, in which the lateral faces are inclined medioventrally, and the ventral surface of the centrum is relatively narrower. The latter condition is frequent among rinconsaurians.

251. Anterior and middle caudal centra, ventral surface: without groove or hollow (0); with groove (1); with hollow divided by a longitudinal septum (2) (Modified from Wilson, 2002: character 132). We added a state to consider the diversity of ventral surfaces in the anterior and middle caudal centra of derived titanosaurians.

252. Middle caudal centra, articular face shape: amphiplatyan or amphicoelous (0); procoelous/distoplatyan (1); slightly procoelous (2); procoelous (3). (González Riga et al., 2009).

253. Posteriormost anteriors and middle caudal vertebrae, location of the neural arches: over the midpoint of the centrum with approximately subequal amounts of the centrum exposed at either end (0); on the anterior half of the centrum (1). (Upchurch et al., 2004: character 185).

254. Anterior caudal vertebrae, anterior face of the centrum strongly inclined anteriorly: straight (vertical) (0); slightly inclined anteroventrally (1); strongly inclined anteroventrally (2). (Modified from Santucci and Arruda Campos, 2011: character 256). Among rinconsaurians the anteroventral inclination of the anterior face of the caudal centra is frequent. However, in *Uberabatitan* and *Trigonosaurus* the

inclination is subtle, whereas in *Aeolosaurus* and *Punatitan*, the anterior surfaces are strongly inclined. The new state allows better separating these differences.

255. Middle caudal vertebrae, with the anterior face strongly inclined anteriorly: absent (0); present (1). (Carballido et al., 2017).

256. Middle caudal vertebrae, height of the pedicels below the prezygapophysis: low with curved anterior edge of the pedicel (0); high with vertical anterior edge of the pedicel (1). (Carballido et al., 2012).

257. Middle caudal vertebrae, orientation of the neural spines: anteriorly (0); vertical (1); slightly directed posteriorly (2); strongly directed posteriorly (3). (Modified from Wilson, 2002: character 133).

258. Posterior caudal vertebrae, neural spine strongly displaced posteriorly: absent (0); present (1). (Carballido et al., 2012).

259. Middle caudal vertebrae, ratio of centrum length to centrum height: less than 2, usually 1.5 or less (0); 2 or higher (1). (Upchurch et al., 2004: character 179).

260. Anterior-posterior caudal vertebrae (those with still well-developed neural spine), neural spine orientation: vertical (0); slightly directed posteriorly (1); strongly directed posteriorly (2). (Carballido et al., 2012).

261. Posterior caudal centra, articular face shape: amphyplatic (0); procoelous (1); opisthocoelous (2). (Modified from González Riga et al., 2009).

262. Posterior caudal centra, shape: cylindrical (0); dorsoventrally flattened, breadth at least twice height (1). (Wilson, 2002: character 135).

263. Posterior caudal vertebrae, ratio of length to height: less than 5, usually 3 or less (0); 5 or higher (1). (Upchurch et al., 2004: character 180).

264. Distalmost caudal centra, articular face shape: platycoelous (0); biconvex (1). (Wilson, 2002: character 136).

265. Distalmost biconvex caudal centra, number: 10 or fewer (0); more than 30 (1). (Wilson, 2002: character 137).

266. Distalmost biconvex caudal centra, length-to height ratio: less than 4 (0); greater than 5 (1). (Wilson, 2002: character 138).

267. Forked chevrons with anterior and posterior projections: absent (0); present (1). (Wilson, 2002: character 143).

268. Forked chevrons, distribution: distal tail only (0); throughout middle and posterior caudal vertebrae (1). (Wilson, 2002: character 144).

269. Chevrons, crus bridging dorsal margin of haemal canal: present (0); absent (1). (Wilson, 2002: character 145).

270. Chevron haemal canal, depth: short, approximately 25% (0); or long, approximately 50% chevron length (1). (Wilson, 2002: character 146).

271. Chevrons: persisting throughout at least 80% of tail (0); disappearing by caudal 30 (1). (Wilson, 2002: character 147).

272. Posterior chevrons, distal contact: fused (0); unfused (open) (1). (Wilson, 2002: character 148).

Scapular girdle

273. Posture: bipedal (0); columnar, obligatory quadrupedal posture (1). (Wilson, 2002: character 149).

274. Scapular acromion process, size: Narrow (0); broad, width more than 150% minimum width of blade (1). (Wilson, 2002: character 150).

275. Scapular blade, orientation respect to coracoid articulation: perpendicular (0); forming a 45º angle (1). (Wilson, 2002: character 151).

276. Scapular blade, distal expansion: absent (0); present (1). This character was introduced for recognizing those sauropods which scapular blade is not markedly expanded distally. The third state is recognized in several sauropods, such as *Patagotitan*, *Alamosaurus*, *Rinconsaurus*. (Carballido et al., 2017).

277. Scapular blade, shape: acromial edge not expanded (both edges are running parallel to each other) (0); rounded expansion on acromial side (1); racquet-shaped (2): marked distal expansion due to the posterodorsal orientation of the dorsal edge (3). (Wilson, 2002: character 152; as modified by Carballido et al., 2017: character 277).

278. Scapula, acromion process dorsal margin: concave or straight (0); with V-shaped concavity (1); with U-shaped concavity (2). (Sereno, 2007: character 88).

279. Scapula, highest point of the dorsal margin of the blade: lower than the dorsal margin of the proximal end (0); at the same height than the dorsal margin of the proximal end (1); higher than the dorsal margin of the proximal end (2). (Carballido et al., 2012, from Mannion, 2009).

280. Scapula, development of the acromion process: undeveloped (0); well developed (1). (Carballido et al., 2012).

281. Scapular length/minimum blade breadth: 5.5 or less (0); 5.5 or more (1). (Carballido et al., 2012).

282. Scapula, ventral margin with a well-developed ventromedial process: absent (0); present (1). (Carballido et al., 2011).

283. Scapular, acromial process position: lies nearly glenoid level (0); lies nearly midpoint scapular body (1). (Carballido et al., 2012).

284. Scapular acromion length: less than 1/2 scapular length (0); at least 1/2 scapular length (1). (Mannion et al., 2012: character168).

285. Glenoid scapular orientation: relatively flat or laterally facing (0); strongly bevelled medially (1). (Wilson, 2002: character 153).

286. Scapular blade, cross-sectional shape at base: flat or rectangular (0); D-shaped (1). (Wilson, 2002: character 154).

287. Coracoid, proximodistal length: less than the length of scapular articulation (0); approximately twice the length of scapular articulation (1). (Wilson, 2002: character 155).

288. Coracoid, anteroventral margin shape: rounded (0); rectangular (1). (Wilson, 2002: character 156).

289. Dorsal margin of the coracoid in lateral view: reaches or surpasses the level of the dorsal margin of the scapular expansion (0); lies below the level of the scapular proximal expansion and separated from the latter by a V-shaped notch (1). (Upchurch et al., 2004: character 207).

290. Coracoid, infraglenoid deep groove: absent (0); present (1). (D’Emic, 2012: character 76)

291. Coracoid, infraglenoid lip: absent (0); present (1). (Wilson, 2002: character 157).

292. Sternal plate, shape: posterolateral margin curved (0); posterolateral margin expanded as a corner (1). (D’Emic, 2012: character 76).

293. Sternal plate, shape: oval (0); crescentic (1). (Wilson, 2002: character 158).

294. Prominent posterolateral expansion of the sternal plate producing a kidney-shaped profile in dorsal view: absent (0); present (1). (Upchurch et al., 2004: character 211).

295. Prominent parasagital oriented ridge on the dorsal surface of the sternal plate: absent (0); present (1). (Upchurch et al., 2004: character 212).

296. Ridge on the ventral surface of the sternal plate: absent (0); present (1). (Upchurch et al., 2004: character 213).

297. Ratio of maximum length of sternal plate to the humerus length: less than 0.75, usually less than 0.65 (0); greater than 0.75 (1). (Upchurch et al., 2004: character 209)

Forelimb

298. Humerus, strong posterolateral bulge around the level of the deltopectoral crest: absent (0); present (1). (D'Emic, 2012: character 80).

299. Humerus, radial and ulnar condyles shape: radial condyle divided on anterior face by a notch (0); undivided (1). (D'Emic, 2012: character 83).

300. Humerus-to-femur ratio: less than 0.60 (0); 0.60 to 0.69 (1); 0.70 to 0.90 (2); greater than 0.90 (3) (Modified from Upchurch et al., 2004: character 216 and Carballido et al., 2017: character 300). We split character state 1 in two, because there are noticeable differences between the humerus/femur proportions in the saltasaurines (0.60 to 0.69) and colossosaurian titanosaurians (0.70 to 0.90).

301. Humeral deltopectoral attachment, development: prominent (0); reduced to a low crest or ridge (1). (Wilson, 2002: character160).

302. Humeral deltopectoral crest, shape: relatively narrow throughout length (0); markedly expanded distally (1). (Wilson, 2002: character161).

303. Humeral midshaft cross-section, shape: circular (0); elliptical (1). (Mannion et al, 2012: character 170).

304. Humerus, RI (*sensu* Wilson and Upchurch, 2003): gracile (less than 0.27) (0); medium (0.28-0.32) (1); robust (more than 0.33) (2). (Carballido et al., 2012).

305. Humeral distal condyles, articular surface shape: restricted to distal portion of humerus (0); exposed on anterior portion of humeral shaft (1). (Wilson, 2002: character 163).

306. Humeral distal condyle, shape: divided (0); flat (1). (Wilson, 2002: character 164).

307. Humeral, lateral margin: medially deflected (0); almost straight until the half length or even more (1); almost straight until the proximal third of the total length of the humerus (2). (Carballido et al., 2012).

308. Humeral proximolateral corner, shape: rounded, the dorsal surface is well convex (0); pronounced / square, the dorsal surface low, almost flat (1). (Wilson, 2002: character 159).

309. Ulnar proximal condyle, shape: subtriangular (0); triradiate, with deep radial fossa (1). (Wilson, 2002: character 165).

310. Ulnar proximal condylar processes, relative lengths: subequal (0); unequal, anterior arm longer (1). (Wilson, 2002: character 166).

311. Ulnar olecranon process, development: prominent, projecting above proximal articulation (0); rudimentary, level with proximal articulation (1). (Wilson, 2002: character 167).

312. Ulna, length-to-proximal breadth ratio: gracile (0); stout (1). (Wilson, 2002: character 168).

313. Radial distal condyle, shape: round (0); subrectangular, flattened posteriorly and articulating in front of ulna (1). (Wilson, 2002: character 169).

314. Radius, distal breadth: slightly larger than midshaft breadth (0); approximately twice midshaft breadth (1). (Wilson, 2002: character 170).

315. Radius, distal condyle orientation: perpendicular to long axis of shaft (0); bevelled approximately 20º proximolaterally relative to long axis of shaft (1). (Wilson, 2002: character 171).

316. Carpal bones, number: 3 or more (0); 2 or fewer (1). (Wilson, 2002: character 173).

317. Carpal bones, shape: round (0); block-shaped, with flattened proximal and distal surfaces (1). (Wilson, 2002: character 174).

318. Metacarpus, shape: spreading (0); bound, with sub-parallel shafts and articular surfaces that extend half their length (1). (Wilson, 2002: character 175).

319. Metacarpals, shape of proximal surface in articulation: gently curving, forming a 90º arc (0); U-shaped, subtending a 270º arc (1). (Wilson, 2002: character 176).

320. Longest metacarpal-to-radius ratio: close to 0.3 (0); 0.45 or more (1). (Wilson, 2002: character 177).

321. Metacarpal I, length: shorter than metacarpal IV (0); longer than metacarpal IV (1). (Wilson, 2002: character 178).

322. Metacarpal I, distal condyle shape: divided (0); undivided (1). (Wilson, 2002: character 179).

323. Metacarpal I distal condyle, transverse axis orientation: bevelled approximately 20º respect to axis of shaft (0); proximodistally or perpendicular with respect to axis of shaft (1). (Wilson, 2002: character 180).

324. Manual digits II and III, phalangeal number: 2-3-4-3-2 or more (0); reduced, 2-2-2-2-2 or less (1); absent or unossified (2). (Wilson, 2002: character 181).

325. Manual phalanx I.1, shape: rectangular (0); wedge-shaped (1). (Wilson, 2002: character 182).

326. Manual non-ungual phalanges, shape: longer proximodistally than broad transversely (0); broader transversely than long proximodistally (1). (Wilson, 2002: character 183).

Pelvic girdle

327. Pelvis, anterior breadth: narrow, ilia longer anteroposteriorly than distance separating preacetabular processes (0); broad, distance between preacetabular processes exceeds anteroposterior length of ilia (1). (Wilson, 200: character 184).

328. Ilium, ischial peduncle size: large, prominent (0); low, rounded (1). (Wilson, 2002: character 185).

329. Ilium, dorsal margin shape: flat (0); semicircular (1). (Wilson, 2002: character 186).

330. Iliun, preacetabular ventral margin shape: straight (0), concave (1); with a convex ventral bump (2). (D'Emic, 2012: character 99)

331. Ilium, preacetabular process shape: pointed, arching ventrally (0); semicircular, with posteroventral excursion of cartilage cap (1). (Wilson, 2002: character 188).

332. Ilium, preacetabular process orientation: anterolateral to body axis (0); perpendicular to body axis (1). (Wilson, 2002: character 189).

333. Highest point on the dorsal margin of the ilium: lies caudal to the base of the pubic process (0); lies cranial to the base of the pubic process (1). (Upchurch et al., 2004: character 245).

334. Pubis length respect to ischium: pubis slightly smaller or subequal to ischium (0); pubis larger (120% +) than ischium (1). (Carballido et al., 2012).

335. Pubis, ambiens process development: small, confluent with anterior margin of pubis prominent, (0); projects anteriorly from anterior margin of pubis (1). (Wilson, 2002: character 189).

336. Pubic apron, shape: flat (straight symphysis) (0); canted anteromedially (gentle S-shaped symphysis) (1). (Wilson, 2002: character 190).

337. Puboischial contact, length: approximately one third total length of pubis (0); one half total length of pubis (1). (Wilson, 2002: character 191).

338. Ischium, acetabular articular surface: maintains approximately the same transverse width throughout its length (0); is transversely narrower in its central portion and strongly expanded as it approaches the iliac and pubic articulations (1). (Mannion et al., 2012: character 180).

339. Ischium, iliac peduncle with constriction or "neck": absent (0); present (1). (Whitlock, 2011: character 173).

340. Ischium, elongate muscle scar on proximal end: absent (0); present (1). (Whitlock, 2011: character 174).

341. Ischial blade, shape: emarginate distal to pubic peduncle (0); no emargination distal to pubic peduncle (1). (Wilson, 2002: character 193).

342. Ischia pubic articulation: less or equal to the anteroposterior length of pubic pedicel (0); greater than the anteroposterior length of pubic pedicel (1). (Salgado et al., 1997).

343. Ischia, anteroposterior pubic pedicel width divided the total length of the ischium: less than 0.5 (0); 0.5 or larger (1). (Carballido et al., 2012).

344. Ischial distal shaft, shape: triangular, depth of ischial shaft increases medially (0); blade-like, medial and lateral depths subequal (1). (Upchurch et al., 2004: character 194).

345. Ischial distal shafts, cross-sectional shape: V-shaped, forming an angle of nearly 50º with each other (0); flat, nearly coplanar (1). (Wilson, 2002: character 195).

346. Ischia, distal end: is only slightly expanded (0); is strongly expanded dorsoventrally (1). (Upchurch, 1998: character 183).

347. Ischium, angle formed between the shaft and the acetabular line: forming an almost right angle (80-110°) (0) or; a close angle (less than 70°) (1). (Carballido et al., 2012).

348. Ischial tuberosity: absent (0); present (1). The tuberosity, noted by Otero (2010) for the ischium of *Neuquensaurus* (Otero, 2010: Fig. 8) and is present in other taxa, such as *Patagotitan*, *Bonitasaura*, *Futalognkosaurus*, and *Alamosaurus*. (Carballido et al., 2017).

Hind limb

349. Femur, longitudinal ridge on the anterior face: absent (0); present (1). (D'Emic, 2012: character 107).

350. Femur, fibular condyle: well developed, having a similar height than the tibial one (0); much shorter than the tibial condyle (1). The fibular condyle of *Patagotitan* and *Bonitasaura* is reduced in its posterior projection respect to that of most other sauropods, which fibular and tibial condyles are almost equally posteriorly projected. (Carballido et al., 2017).

351. Femur, epicondyle development: well developed (0); reduced, almost absent (1). In *Patagotitan* the epicondyle is extremely developed and notorious in posterior and distal view, as a minor step laterally projected. In contrast in some titanosaurians the epicondyle is almost imperceptible, as is the case of *Dreadnoughtus*, *Opisthocoelicaudia*, *Neuquensaurus* and *Saltasaurus*. (Carballido et al., 2017).

352. Femur, fourth trochanter position: almost at the half of the femur (0); in the proximal third of the femur (1). The fourth trochanter of *Patagotitan* is positioned around the proximal third of the total femur length, similar to the position observed in *Futalognkosaurus*, *Bonitasaura*, and some other non-Lognkosauria as *Rapetosaurus*, *Saltasaurus* and *Neuquensaurus*. In contrast the fourth trochanter of

most sauropods is around the half of the total femur length, being even lower in *Opisthocoelicaudia*. (Carballido et al., 2017).

353. Femur, fourth trochanter development: prominent (0); reduced to crest or ridge (1); extremely reduced (2). (Modified from Wilson, 2002: character 196, following to Whitlock, 2011: character 186).

354. Femur, lesser trochanter: present (0); absent (1). (Wilson, 2002: character 197).

355. Femur midshaft, transverse diameter: subequal to anteroposterior diameter (0); 125- 150% anteroposterior diameter (1); at least 185% anteroposterior diameter (2). (Wilson, 2002: character 198).

356. Femur, lateral bulge (marked by the lateral expansion and a dorsomedial orientation of the laterodorsal margin of the femur, which starts below the femur head ventral margin): absent (0); present (1). (Salgado et al., 1997).

357. Femur, pronounced ridge on posterior surface between greater trochanter and head: absent (0); present (1). (Whitlock, 2011: character 181).

358. Femur head position: perpendicular to the shaft, rises at the same level as the greater trochanter (0); dorsally directed, rises well above the level of the greater trochanter (1). (Modified from Upchurch et al., 2004: character 263).

359. Femur, distal condyles relative transverse breadth: subequal (0); tibial much broader than fibular (1). (Wilson, 2002: character 2000).

360. Femur, distal condyles orientation: perpendicular or slightly bevelled dorsolaterally (0); or bevelled dorsomedially approximately 10º relative to femoral shaft (1). (Wilson, 2002: character 201).

361. Femur, distal condyles articular surface shape: restricted to distal portion of femur (0); expanded onto anterior portion of femoral shaft (1). (Wilson, 2002: character 202).

362. Situation of the femoral fourth trochanter: on the caudal surface of the shaft, near the midline (0); on the caudomedial margin of the shaft (1). (Upchurch et al., 2004: character 268).

363. Tibial proximal condyle, shape: narrow, long axis anteroposterior (0); expanded transversely, condyle subcircular (1). (Wilson, 2002: character 203).

364. Tibial cnemial crest, orientation: projecting anteriorly (0); or laterally (1). (Wilson, 2002: character 204).

365. Tibia, distal breadth: approximately 125% (0); more than twice midshaft breadth (1). (Wilson, 2002: character 205).

366. Tibial distal posteroventral process, size: broad transversely, covering posterior fossa of astragalus (0); shortened transversely, posterior fossa of astragalus visible posteriorly (1). (Wilson, 2002: character 206).

367. Fibula, proximal tibial scar, development: not well-marked (0); well-marked and deepening anteriorly (1). (Wilson, 2002: character 207).

368. Fibula, lateral trochanter: absent (0); present (1). (Wilson, 2002: character 208).

369. Fibular distal condyle, size: subequal to shaft (0); expanded transversely, more than twice midshaft breadth (1). (Wilson, 2002: character 209).

370. Fibular, proximal end, anterior crest: absent or poorly-developed (0); well-developed creating an interlocking proximal crus (1). (D'Emic, 2012: character 111).

371. Fibula, shaft shape: straight, or slightly sigmoidal (0); sigmoid, such that the proximal and distal faces are angled relative to midshaft (1). (D'Emic, 2012: character 113).

372. Astragalus, shape: at least 1.5 times wider than anteroposteriorly long (0); anteroposterior and transverse dimensions subequal (1). (D'Emic, 2012: character 115).

373. Astragalus, shape: rectangular (0); wedge shaped, with reduced anteromedial corner (1). (Wilson, 2002: character210).

374. Astragalus, fibular facet: faces laterally (0); faces posterolaterally, anterior margin visible in posterior view (1). (Whitlock, 2011: character 186).

375. Astragalus, foramina at base of ascending process: present (0); absent (1). (Wilson, 2002: character 211).

376. Astragalus, ascending process length: limited to anterior two-thirds of astragalus (0); extending to posterior margin of astragalus (1). (Wilson, 2002: character 212).

377. Astragalus, posterior fossa shape: undivided (0); divided by vertical crest (1). (Wilson, 2002: character 213).

378. Astragalus, transverse length: 50% more than (0); or subequal to proximodistal height (1). (Wilson, 2002: character 214).

379. Calcaneum: present (0); absent or unossified (1). (Wilson, 2002: character 215).

380. Distal tarsals 3 and 4: present (0); absent or unossified (1). (Wilson, 2002: character 216).

381. Metatarsus, posture: bound (0); spreading (1). (Wilson, 2002: character 217).

382. Metatarsal I proximal condyle, transverse axis orientation: perpendicular to (0); angled ventromedially approximately 15º to axis of shaft (1). (Wilson, 2002: character 218).

383. Metatarsal I distal condyle, transverse axis orientation: perpendicular to (0); angled dorsomedially to axis of shaft (1). (Wilson, 2002: character 219).

384. Metatarsal III length divided by metatarsal I length: less than 1.3 (0); more than 1.3 (1). (González Riga et al., 2016: character 331).

385. Longest metatarsal: metatarsal III (0); metatarsal IV (1). (González Riga et al., 2016: character 334).

386. Metatarsal I distal condyle, posterolateral projection: absent (0); present (1). (Wilson, 2002: character 220).

387. Metatarsal I, minimum shaft width: less than that of metatarsals II-IV (0); or greater than that of metatarsals II-IV (1). (Wilson, 2002: character 221).

388. Metatarsal I and V proximal condyle, size: smaller than (0); or subequal to those of metatarsals II and IV (1). (Wilson, 2002: character 222).

389. Metatarsal III length: more than 30% (0); or less than 25% that of tibia (1). (Wilson, 2002: character 223).

390. Metatarsals III and IV, minimum transverse shaft diameters: subequal to (0); or less than 65% that of metatarsals I or II (1). (Wilson, 2002: character 224).

391. Metatarsal IV, proximomedial end, shape: flat or slightly concave (0); possesses a distinct embayment (1). (D'Emic, 2012: character 117).

392. Metatarsal IV, distal end, orientation: roughly perpendicular to long axis of bone (0); bevelled upwards medially (1). (D'Emic, 2012: character 118).

393. Metatarsal V, length: shorter than (0); or at least 70% length of metatarsal IV (1). (Wilson, 2002: character 225).

394. Pedal non-ungual phalanges, shape: longer proximodistally than broad transversely (0); broader transversely than long proximodistally (1). (Wilson, 2002: character 226).

395. Pedal digits II-IV, penultimate phalanges, development: subequal in size to more proximal phalanges (0); rudimentary or absent (1). (Wilson, 2002: character 227).

396. Pedal unguals, orientation: aligned with (0); or deflected lateral to digit axis (1). (Wilson, 2002: character 228).

397. Pedal digit I ungual, length relative to pedaldigit II ungual: subequal (0); 25% larger than that of digit II (1). (Wilson, 2002: character 229).

398. Pedal digit I ungual, length: shorter (0); or longer than metatarsal I (1). (Wilson, 2002: character 230).

399. Pedal ungual I, shape: broader transversely than dorsoventrally (0); sickle-shaped, much deeper dorsoventrally than broad transversely (1). (Wilson, 2002: character 231).

400. Pedal ungual II-III, shape: broader transversely than dorsoventrally (0); sickleshaped, much deeper dorsoventrally than broad transversely (1). (Wilson, 2002: character 232).

401. Pedal digit IV ungual, development: subequal in size to unguals of pedal digits II and III (0); rudimentary or absent (1). (Wilson, 2002: character 233).

402. Unguals of pedal digit II and III, proximal dimensions: as broad as deep (0); significantly broader than deep (1). (Allain and Aquesbi, 2008: character 253).

403. Number of phalanges in pedal digit II: 3 (0); 2 (1). (González Riga et al., 2016: character 348).

404. Number of phalanges in pedal digit III: 4 (0); 3 (1). (González Riga et al., 2016: character 349).

405. Number of phalanges in pedal digit IV: 3 or more (0); 2 (1); 1 (2). (González Riga et al., 2016: character 350)

406. Postorbital, excluded from the infratemporal fenestra due to the articulation of the jugal with the squamosal: absent (0), present (1). (Canudo et al., 2018).

407. Squamosal, ventral shape: thin (0); broad (1). (Canudo et al., 2018).

408. Preantorbital fenestra development: small, differentiated from the posterior maxillary foramen in its direction (see Wilson and Sereno, 1998) (0); laterally opened middle sized fenestra (1); laterally opened large fenestra (2). (Canudo et al., 2018).

409. Mid- and posterior dorsal neural arches, centroprezygapophyseal fossa depth: shallow or absent (0); deep, passing nearly all the way through the neural arch. (Wilson and Allain, 2015: character 101).

410. Mid- posterior dorsal vertebrae, parapophysis, position with respect to prezygapophyses: at the same level or below (0); well above (1). (Wilson and Allain, 2015: character 100).

411. Posterior dorsal neural arches, centroprezygapophyseal lamina (CPRL), shape: single (0); divided (1). (Wilson and Allain, 2015: character 107).

412. Posterior dorsal neural arches, spinoparapophyseal lamina (SPPL): absent (0); present (1). (Wilson and Allain, 2015: character 109).

413. Middle caudal vertebrae, prezygapophyses orientation: anterodorsally oriented (around 45 degrees) (0); anteriorly oriented (nearly horizontal) (1). (Canudo et al., 2018).

414. Scapular acromion, ventral process: absent (0), present (1). (Carballido et al., 2020).

415. Ilium, postacetabular posteroventral edge: open concave (0); U-shaped notch (1); horizontal and low V-shaped notch (2). (Carballido et al., 2020).

416. Pubis, ischiadic articular surface: continuous without marked angle change (0); marked step formed by a proximal posterior directed surface and a more distal posterodorsally oriented surface (1). (Carballido et al., 2020).

417. Pubis, proximal symphysis: merges with the pubic shaft (0); forms a marked ventromedially directed process (1). (Carballido et al., 2020).

Characters added

418. Posteriormost anterior and middle caudal centra, proportions: as high as wide (high) (0), wider than high (depressed) (1). (Modified from Salgado et al., 1997: character 34).

419. Middle caudal vertebrae, anterodorsal end of the neural spine located posteriorly with respect to anterior border of the postzygapophyses: absent (0); present (1) (Salgado et al., 1997: character 10).

420. Middle caudal vertebrae, length proportions of prezygapophyses with respect to the centrum length: shorter than 40% (0); between 40 and 50% (1); longer than 50% (2). (Modified from Salgado et al., 2014: character 54).

421. Posteriormost anterior and middle caudal vertebrae, prezygapophyses distally expanded dorsoventrally: absent (0); present (1). (Santucci and Arruda-Campos, 2011).

422. Quadrate, articular condyle: undivided (0); anteriorly divided (1); posteriorly divided (2); entirely divided by a sulcus, in a medial and lateral condyle (3). New.

References

Allain, R., Aquesbi, N., 2008. Anatomy and phylogenetic relationships of *Tazoudasaurus naimi* (Dinosauria, Sauropoda) from the late Early Jurassic of Morocco. Geodiversitas 30, 345–424.

Allen, J.R.L., 1983. Studies in fluviatile sedimentation: bars, bar-complexes and sandstone sheets (low sinuosity braided streams) in the Brownstones (L. Devonian), Welsh Borders. Sedimentary Geology 33, 237–293.

Apesteguía, S., 2004. *Bonitasaura salgadoi* gen. et sp. nov.: a beaked sauropod from the Late Cretaceous of Patagonia. Naturwissenschaften 91(10), 493–497.

Arcucci, A.B., Marsicano, C.A., Coria, R.A., 2005. Una nueva localidad fosilífera en el Cretácico de la Precordillera de La Rioja. Ameghiniana 42, 60R.

Bandeira, K.L.N., Simbras, F.M., Machado, E.B., De Almeida Campos, D., Oliveira, G.R., Kellner, A.W.A., 2016. A new giant Titanosauria (Dinosauria: Sauropoda) from the Late Cretaceous Bauru Group, Brazil. PLoS ONE 11, 1–25. doi:10.1371/journal.pone.0163373

Behrensmeyer, A.K., Hook, R.W., 1992. Paleoenvironmental contexts and taphonomic modes, in: Behrensmeyer, A., Damuth, J., Di Michele, W., Potts, R., Sues, H.-D., Wings, S. (Eds.), Terrestrial Ecosystems through Time: Evolutionary Paleoecology of Terrestrial Plants and Animals. University of Chicago Press, Chicago, pp. 15–93.

Bonaparte, J.F., Coria, R.A., 1993. Un nuevo y gigantesco saurópodo titanosaurio de la Formación Río Limay (Albiano-Cenomaniano) de la Provincia del Neuquén, Argentina. Ameghiniana 30, 271–282.

Bonaparte, J.F., Powell, J.E., 1980. A continental assemblage of tetrapods from the Upper Cretaceous beds of El Brete, northwest Argentina (Sauropoda-Coelurosauria-Carnosauria-Aves), Table ronde internationale du Ecosystèmes Continentaux du Mésozoïque.

Bracaccini, O., 1946. Contribución al conocimiento geológico de la Precordillera Sanjuanina-Mendocina. Boletín de Informaciones Petroleras1 258, 81–105.

Bridge, J.S., 2003. Rivers and floodplains: forms, processes and sedimentary record. Wiley-Blackwell.

Burns, C.E., Mountney, N.P., Hodgson, D.M., Colombera, L., 2017. Anatomy and dimensions of fluvial crevasse-splay deposits: Examples from the Cretaceous Castlegate Sandstone and Neslen Formation, Utah, U.S.A. Sedimentary Geology 351, 21–35. doi:10.1016/j.sedgeo.2017.02.003

Calvo, J.O., González Riga, B.J., 2003. *Rinconsaurus caudamirus* gen. et sp. nov., a new titanosaurid (Dinosauria, Sauropoda) from the Late Cretaceous of Patagonia, Argentina. Revista Geológica de Chile 30, 333–353.

Calvo, J.O., González Riga, B.J., Porfiri, J.D., 2007a. A new titanosaur sauropod from the Late Cretaceous of Neuquén, Patagonia, Argentina. Arquivos do Museu

Nacional 65, 485–504.

Calvo, J.O., Porfiri, J.D., González-Riga, B.J., Kellner, A.W. a, 2007b. A new Cretaceous terrestrial ecosystem from Gondwana with the description of a new sauropod dinosaur. Anais da Academia Brasileira de Ciências 79, 529–41.

Campos, D.A., Kellner, A.W.A., Bertini, R.J., Santucci, R.M., 2005. On a titanosaurid (Dinosauria, Sauropoda) vertebral column from the Bauru Group, Late Cretaceous of Brazil. Arquivos do Museu Nacional, Rio do Janeiro 63, 565–596.

Canudo, J.I., Carballido, J.L., Garrido, A., Salgado, L., 2018. A new rebbachisaurid sauropod from the Aptian-Albian, Lower Cretaceous Rayoso Formation, Neuquén, Argentina. Acta Palaeontologica Polonica 63, 679–691. doi:10.4202/app.00524.2018

Carballido, J.L., Pol, D., Cerda, I., Salgado, L., 2011. The osteology of *Chubutisaurus insignis* del Corro, 1975 (Dinosauria: Neosauropoda) from the ‘middle’ Cretaceous of central Patagonia, Argentina. Journal of Vertebrate Paleontology 31, 93–110. doi:10.1080/02724634.2011.539651

Carballido, J.L., Pol, D., Otero, A., Cerda, I.A., Salgado, L., Garrido, A.C., Ramezani, J., Cúneo, N.R., Krause, J.M., 2017. A new giant titanosaur sheds light on body mass evolution among sauropod dinosaurs. Proceedings of the Royal Society B: Biological Sciences 284, 20171219. doi:10.1098/rspb.2017.1219

Carballido, J.L., Salgado, L., Pol, D., Canudo, J.I., Garrido, A., 2012. A new basal rebbachisaurid (Sauropoda, Diplodocoidea) from the Early Cretaceous of the Neuquén Basin; evolution and biogeography of the group. Historical Biology 24, 631–654. doi:10.1080/08912963.2012.672416

Carballido, J.L., Scheil, M., Knötschke, N., Sander, P.M., 2020. The appendicular skeleton of the dwarf macronarian sauropod *Europasaurus holgeri* from the Late Jurassic of Germany and a re-evaluation of its systematic affinities. Journal of Systematic Palaeontology 18, 739–781. doi:10.1080/14772019.2019.1683770

Cerda, I.A., Paulina Carabajal, A., Salgado, L., Coria, R.A., Reguero, M.A., Tambussi, C.P., Moly, J.J., 2012. The first record of a sauropod dinosaur from Antarctica. Naturwissenschaften 99, 83–7. doi:10.1007/s00114-011-0869-x

Chaía, T., 1990. Registro del Cenoniano lacustre en la provincia de San Juan, in: 1° Congreso Uruguayo de Geología, Resúmenes Ampliados. Montevideo, pp. 33–36.

Ciccioli, P.L., Ballent, S., Tedesco, A.M., Barreda, V., Limarino, C.O., 2005. Hallazgo de depósitos Cretácicos en la Precordillera de La Rioja (Formación Ciénaga del Río Huaco). Revista de la Asociación Geológica Argentina 60, 122–131.

Coria, R.A., Filippi, L.S., Chiappe, L.M., 2013. *Overosaurus paradasorum* gen. et sp. nov., a new sauropod dinosaur (Titanosauria: Lithostrotia) from the Late Cretaceous of Neuquén, Patagonia, Argentina. Zootaxa 3683, 357–376.

Coughlin, T.J., 2000. Linked Orogen-Oblique Fault Zones in the Central Andes: the basis of a new model for Andean orogenesis and metallogenesis. University of Queensland, Brisbane.

Curry-Rogers, K.A., 2005. Titanosauria: a phylogenetic overview, in: Curry-Rogers, K.A., Wilson, J.A. (Eds.), The Sauropods: Evolution and Paleobiology. University of California Press, pp. 50–103.

D'Emic, M.D., 2012. The early evolution of titanosauriform sauropod dinosaurs. Zoological Journal of the Linnean Society 166, 624–671. doi:10.1111/j.1096-3642.2012.00853.x

Faria, C.C. de J., González Riga, B., Candeiro, C.R. dos A., Marinho, T. da S., Ortiz David, L., Simbras, F.M., Castanho, R.B., Muniz, F.P., Gomes da Costa Pereira, P.V.L., 2015. Cretaceous sauropod diversity and taxonomic succession in South America. Journal of South American Earth Sciences 61, 154–163. doi:10.1016/j.jsames.2014.11.008

Fosdick, J.C., Reat, E.J., Carrapa, B., Ortiz, G., Alvarado, P.M., 2017. Retroarc basin reorganization and aridification during Paleogene uplift of the southern central Andes. Tectonics 36, 493–514. doi:10.1002/2016TC004400

Fowler, D.W., Hall, L.E., 2011. Scratch-digging sauropods, revisited. Historical Biology 23, 27–40. doi:10.1080/08912963.2010.504852

Franco-Rosas, A.C., Salgado, L., Rosas, C.F., de Souza Carvalho, I., 2004. Nuevos materiales de titanosaurios (Sauropoda) en el Cretácico Superior de Mato Grosso, Brasil. Revista Brasileira de Paleontologia 7, 329–336. doi:10.4072/rbp.2004.3.04

Goloboff, P.A., Farris, J.S., Nixon, K.C., 2008. TNT, a free program for phylogenetic analysis. Cladistics 24, 774–786.

González Riga, B.J., 2003. A new titanosaur (Dinosauria, Sauropoda) from the Upper Cretaceous of Mendoza Province, Argentina. Ameghiniana 40, 155–172.

González Riga, B.J., Lamanna, M.C., Ortiz David, L.D., Calvo, J.O., Coria, J.P., 2016. A gigantic new dinosaur from Argentina and the evolution of the sauropod hind foot. Scientific Reports 6, 19165. doi:10.1038/srep19165

González Riga, B.J., Mannion, P.D., Poropat, S.F., Ortiz David, L.D., Coria, J.P., 2018. Osteology of the Late Cretaceous Argentinean sauropod dinosaur *Mendozasaurus neguyelap*: Implications for basal titanosaur relationships. Zoological Journal of the Linnean Society 184, 136–181. doi:10.1093/zoolinnean/zlx103

González Riga, B.J., Ortiz David, L., 2014. A new titanosaur (Dinosauria, Sauropoda) from the Upper Cretaceous (Cerro Lisandro Formation) of Mendoza province, Argentina. Ameghiniana 51, 3–25. doi:10.5710/AMEGH.26.12.1013.1889

González Riga, B.J., Previtera, E., Pirrone, C.A., 2009. Malarguesaurus florenciae gen. et sp. nov., a new titanosauriform (Dinosauria, Sauropoda) from the Upper Cretaceous of Mendoza, Argentina. Cretaceous Research 30, 135–148. doi:10.1016/j.cretres.2008.06.006

Gorscak, E., O'Connor, P.M., 2016. Time-calibrated models support congruency between Cretaceous continental rifting and titanosaurian evolutionary history. Biology Letters 12, 20151047. doi:10.1098/rsbl.2015.1047

Harris, J.D., 2006. Cranial osteology of *Suuwassea emiliae* (Sauropoda: Diplodocoidea:

Flagellicaudata) from the Upper Jurassic Morrison Formation of Montana, USA. Journal of Vertebrate Paleontology 26, 88–102. doi:10.1671/0272-4634(2006)26[88:COOSES]2.0.CO;2

Hechenleitner, E.M., Fiorelli, L.E., Martinelli, A.G., Grellet-Tinner, G., 2018. Titanosaur dinosaurs from the Upper Cretaceous of La Rioja province, NW Argentina. Cretaceous Research 85, 42–59. doi:10.1016/j.cretres.2018.01.006

Hechenleitner, E.M., Grellet-Tinner, G., Fiorelli, L.E., 2015. What do giant titanosaur dinosaurs and modern Australasian megapodes have in common? PeerJ 3, e1341. doi:10.7717/peerj.1341

Hubert, J.F., Hyde, M.G., 1982. Sheet-flow deposits of graded beds and mudstones on an alluvial sandflat-playa system: Upper Triassic Blomidon redbeds, St Mary's Bay, Nova Scotia. Sedimentology 29, 457–474. doi:10.1111/j.1365-3091.1982.tb01730.x

Jordan, T.E., Isacks, B.L., Allmendinger, R.W., Brewer, J.A., Ramos, V.A., Ando, C.J., 1983. Andean tectonics related to geometry of subducted Nazca plate. Geological Society of America Bulletin 94, 341. doi:10.1130/0016-7606(1983)94<341:ATRTGO>2.0.CO;2

Kellner, A.W.A., de Azevedo, S.A.K., 1999. A new sauropod dinosaur (Titanosauria) from the Late Cretaceous of Brazil, in: Tomida, Y., Rich, T.H., Vickers-Rich, P. (Eds.), Proceedings of the Second Gondwanan Dinosaur Sympasium. National Science Museum Monographs, No. IS, Tokio, pp. 111–142.

Lacovara, K.J., Lamanna, M.C., Ibiricu, L.M., Poole, J.C., Schroeter, E.R., Ullmann, P. V., Voegele, K.K., Boles, Z.M., Carter, A.M., Fowler, E.K., Egerton, V.M., Moyer, A.E., Coughenour, C.L., Schein, J.P., Harris, J.D., Martínez, R.D., Novas, F.E., 2014. A gigantic, exceptionally complete titanosaurian sauropod dinosaur from southern Patagonia, Argentina. Scientific Reports 4, 1–9. doi:10.1038/srep06196

Limarino, C.O., Ciccioli, P.L., Krapovickas, V., Benedito, L.D., 2016. Estratigrafía de las sucesiones mesozoicas, paleógenas y neógenas de las quebradas Santo Domingo y el Peñón (Precordillera Septentrional riojana). Revista de la Asociación Geológica Argentina 73, 301–318.

Limarino, C.O., Net, L., Gutiérrez, P., Barreda, V., Caselli, A., Ballent, S., 2000. Definición litoestratigráfica de la Formación Ciénaga del Río Huaco (Cretácico Superior), Precordillera central, San Juan, Argentina. Revista de la Asociación Geológica Argentina 55, 83–99.

Mannion, P.D., Upchurch, P., Mateus, O., Barnes, R.N., Jonese, M.E.H., 2012. New information on the anatomy and systematic position of *Dinheirosaurus lourinhanensis* (Sauropoda: Diplodocoidea) from the Late Jurassic of Portugal, with a review of European diplodocoids. Journal of Systematic Palaeontology 10, 521–551. doi:10.1080/14772019.2011.595432

Martinelli, A., Riff, D., Lopes, R., 2011. Discussion about the occurrence of the genus *Aeolosaurus* Powell 1987 (Dinosauria, Titanosauria) in the Upper Cretaceous of Brazil. Gaea 7, 34–40. doi:10.4013/gaea.2011.71.03

Martínez, R.D.F., Lamanna, M.C., Novas, F.E., Ridgely, R.C., Casal, G.A., Martínez, J.E., Vita, J.R., Witmer, L.M., 2016. A basal lithostrotian titanosaur (Dinosauria: Sauropoda) with a complete skull: implications for the evolution and paleobiology of Titanosauria. Plos One 11, e0151661. doi:10.1371/journal.pone.0151661

Miall, A.D., 1996. The geology of fluvial deposits. Springer, Berlin.

Müller, R.D., Cannon, J., Qin, X., Watson, R.J., Gurnis, M., Williams, S., Pfaffelmoser, T., Seton, M., Russell, S.H.J., Zahirovic, S., 2018. GPlates: Building a Virtual Earth Through Deep Time. Geochemistry, Geophysics, Geosystems 19, 2243–2261. doi:10.1029/2018GC007584

Navarrete, C., Casal, G., Martínez, R., 2011. *Drusilasaura deseadensis* gen. et sp. nov., un nuevo titanosaurio (Dinosauria-Sauropoda), de la Formación Bajo Barreal, Cretácico Superior del Norte de Santa Cruz, Argentina. Revista Brasileira De Paleontologia 14, 1–14. doi:10.4072/rbp.2011.1.01

Novas, F.E., Salgado, L., Calvo, J.O., Agnolin, F.L., 2005. Giant titanosaur (Dinosauria, Sauropoda) from the Late Cretaceous of Patagonia. Revista del Museo Argentino de Ciencias Naturales 7, 37–41.

Pérez, M.., Fernández Seveso, F., Álvarez, L.A., Brison, I.E., 1993. Análisis ambiental y estratigráfico del Paleozoico Superior en el área anticlinal de Huaco, San Juan, Argentina, in: 10° Congrés International de La Stratigraphie et Geólogie Du Carbonifére et Permien, Comptes Rendus, Actas 2. pp. 297–318.

Pianka, E.R.., 1966. Latitudinal gradients in species diversity: a review of concepts. American Naturalist 100, 33–46.

Pol, D., Garrido, A., Cerda, I.A., 2011. A new sauropodomorph dinosaur from the Early Jurassic of Patagonia and the origin and evolution of the sauropod-type sacrum. PloS ONE 6, e14572. doi:10.1371/journal.pone.0014572

Powell, J.E., 1990. *Epachthosaurus sciuttoi* (gen. et sp. nov.) un dinosaurio sauropodo del Cretácico de Patagonia (Provincia de Chubut, Argentina), in: V Congreso Argentino de Paleontología y Bioestratigrafía. pp. 123–128.

Powell, J.E., 2003. Revision of South American titanosaurid dinosaurs: palaeobiological, palaeobiogeographical and phylogenetic aspects. Records of the Queen Victoria Museum 111, 1–173.

Ramos, V.A., 1988. The tectonics of the Central Andes; 30 to 33 S latitude. Special Paper of the Geological Society of America 218, 31–54. doi:10.1130/SPE218-p31

Rauhut, O.W.M., Carballido, J.L., Pol, D., 2015. A diplodocid sauropod dinosaur from the Late Jurassic Canadon Calcareo Formation of Chubut, Argentina. Journal of Vertebrate Paleontology 35. doi:10.1080/02724634.2015.982798

Reat, E.J., Fosdick, J.C., 2018. Basin evolution during Cretaceous-Oligocene changes in sediment routing in the Eastern Precordillera, Argentina. Journal of South American Earth Sciences 84, 422–443. doi:10.1016/j.jsames.2018.02.010

Remes, K., Ortega, F., Fierro, I., Joger, U., Kosma, R., Ferrer, J.M.M., Ide, O.A., Maga, A., 2009. A new basal sauropod dinosaur from the middle Jurassic of Niger and the

early evolution of Sauropoda. PloS ONE 4, e6924. doi:10.1371/journal.pone.0006924

Salgado, L., Azpilicueta, C., 2000. Un nuevo saltasaurino (Sauropoda, Titanosauridae) de la provincia de Río Negro (Formación Allen, Cretácico Superior), Patagonia, Argentina. Ameghiniana 37, 259–264.

Salgado, L., Carvalho, I.D.S., 2008. *Uberabatitan ribeiroi*, a new titanosaur from the Marilia Formation (Bauru Group, Upper Cretaceous), Minas Gerais, Brazil. Palaeontology 51, 881–901. doi:10.1111/j.1475-4983.2008.00781.x

Salgado, L., Coria, R.A., Calvo, J.O., 1997. Evolution of titanosaurid sauropods. I: Phylogenetic analysis based on the postcranial evidence. Ameghiniana 34, 3–32.

Salgado, L., Gallina, P.A., Paulina Carabajal, A., 2014. Redescription of *Bonatitan reigi* (Sauropoda: Titanosauria), from the Campanian–Maastrichtian of the Río Negro Province (Argentina). Historical Biology 27, 1–24. doi:10.1080/08912963.2014.894038

Santucci, R.M., Arruda-Campos, A.C. de, 2011. A new sauropod (Macronaria, Titanosauria) from the Adamantina Formation, Bauru Group, Upper Cretaceous of Brazil and the phylogenetic relationships of Aeolosaurini. Zootaxa 33, 1–33.

Sax, D.F., 2001. Latitudinal gradients and geographic ranges of exotic species: implications for biogeography. Journal of Biogeography 28, 139–150.

Sereno, P.C., 2007. The phylogenetic relationships of early dinosaurs: a comparative report. Historical Biology 19, 145–155. doi:10.1080/08912960601167435

Seton, M., Müller, R.D., Zahirovic, S., Gaina, C., Torsvik, T., Shephard, G., Talsma, A., Gurnis, M., Turner, M., Maus, S., Chandler, M., 2012. Global continental and ocean basin reconstructions since 200Ma. Earth-Science Reviews 113, 212–270. doi:10.1016/j.earscirev.2012.03.002

Silva, J.C.G., Marinho, T.S., Martinelli, A.G., Langer, M.C., 2019. Osteology and systematics of *Uberabatitan ribeiroi* (Dinosauria; Sauropoda): A Late Cretaceous titanosaur from Minas Gerais, Brazil. Zootaxa 4577, 401–438. doi:10.11646/zootaxa.4577.3.1

Tedesco, A.M., Limarino, C.O., Ciccioli, P.L., 2007. Primera edad radimétrica de los depósitos cretácicos de la precordillera central. Revista de la Asociación Geológica Argentina 62, 471–474.

Tunbridge, I.P., 1984. Facies model for a sandy ephemeral stream and clay playa complex; the Middle Devonian Trentishoe Formation of North Devon, UK. Sedimentology 31, 697–715.

Tykoski, R.S., Fiorillo, A.R., 2016. An articulated cervical series of *Alamosaurus sanjuanensis* Gilmore, 1922 (Dinosauria, Sauropoda) from Texas: new perspective on the relationships of North America’s last giant sauropod. Journal of Systematic Palaeontology 2019, 1–26. doi:10.1080/14772019.2016.1183150

Upchurch, P., 1998. The phylogenetic relationships of sauropod dinosaurs. Zoological Journal ofthe Linnean Society 124, 43–103.

Upchurch, P., Barrett, P.M., Dodson, P., 2004. Sauropoda, in: The Dinosauria. pp. 259–324.

Upchurch, P., Barrett, P.M., Galton, P.M., 2007. A phylogenetic analysis of basal sauropodomorph relationships: implications for the origin of sauropod dinosaurs. Special Papers in Palaeontology 57–90.

Vila, B., Galobart, À., Oms, O., Poza, B., Bravo, A.M., 2010a. Assessing the nesting strategies of Late Cretaceous titanosaurs: 3-D clutch geometry from a new megaloolithid eggsite. Lethaia 43, 197–208. doi:10.1111/j.1502-3931.2009.00183.x

Vila, B., Jackson, F.D., Fortuny, J., Sellés, A.G., Galobart, A., 2010b. 3-D modelling of megaloolithid clutches: insights about nest construction and dinosaur behaviour. PloS ONE 5, e10362. doi:10.1371/journal.pone.0010362

Whitlock, J.A., 2011. A phylogenetic analysis of Diplodocoidea (Saurischia: Sauropoda). Zoological Journal of the Linnean Society 161, 872–915. doi:10.1111/j.1096-3642.2010.00665.x

Wilson, J.A., 2002. Sauropod dinosaur phylogeny: critique and cladistic analysis. Zoological Journal of the Linnean Society 136, 217–276.

Wilson, J.A., Allain, R., 2015. Osteology of Rebbachisaurus garasbae Lavocat, 1954, a diplodocoid (Dinosauria, Sauropoda) from the early Late Cretaceous-aged Kem Kem beds of southeastern Morocco. Journal of Vertebrate Paleontology 35, 37–41. doi:10.1080/02724634.2014.1000701

Zaher, H., Pol, D., Carvalho, A.B., Nascimento, P.M., Riccomini, C., Larson, P., Juarez-Valieri, R., Pires-Domingues, R., da Silva, N.J., Campos, D.D.A., 2011. A complete skull of an Early Cretaceous sauropod and the evolution of advanced titanosaurians. PLoS ONE 6, e16663. doi:10.1371/journal.pone.0016663
