# Supplementary material for: Two Late Cretaceous sauropods reveal titanosaurian dispersal across South America
Source: Commun Biol. 2020 Oct 27;3:622. doi: 10.1038/s42003-020-01338-w (PMC7591563; doi:10.1038/s42003-020-01338-w)
Supplement: Supplementary file 2 — Description of Additional Supplementary Files [file 42003_2020_1338_MOESM2_ESM.pdf]

Description of Additional Supplementary Files

File Name: Supplementary Data 1
Description: List of scoring modifications introduced to the dataset of Carballido et al., 2020.

File Name: Supplementary Data 2
Description: Data matrix in .tnt format.
