# Supplementary material for: Two Late Cretaceous sauropods reveal titanosaurian dispersal across South America
Source: Commun Biol. 2020 Oct 27;3:622. doi: 10.1038/s42003-020-01338-w (PMC7591563; doi:10.1038/s42003-020-01338-w)
Supplement: Supplementary file 6 — Reporting Summary [file 42003_2020_1338_MOESM6_ESM.pdf]

nature research

Corresponding author(s): E. Martín Hechenleitner

Last updated by author(s): Aug 27, 2020

Reporting Summary

Nature Research wishes to improve the reproducibility of the work that we publish. This form provides structure for consistency and transparency in reporting. For further information on Nature Research policies, see Authors & Referees and the Editorial Policy Checklist .

Statistics

For all statistical analyses, confirm that the following items are present in the figure legend, table legend, main text, or Methods section.

n/a	Confirmed	
x		The exact sample size (*n*) for each experimental group/condition, given as a discrete number and unit of measurement
x		A statement on whether measurements were taken from distinct samples or whether the same sample was measured repeatedly
x		The statistical test(s) used AND whether they are one- or two-sided<br>*Only common tests should be described solely by name; describe more complex techniques in the Methods section.*
x		A description of all covariates tested
x		A description of any assumptions or corrections, such as tests of normality and adjustment for multiple comparisons
x		A full description of the statistical parameters including central tendency (e.g. means) or other basic estimates (e.g. regression coefficient) AND variation (e.g. standard deviation) or associated estimates of uncertainty (e.g. confidence intervals)
x		For null hypothesis testing, the test statistic (e.g. *F*, *t*, *r*) with confidence intervals, effect sizes, degrees of freedom and *P* value noted<br>*Give P values as exact values whenever suitable.*
x		For Bayesian analysis, information on the choice of priors and Markov chain Monte Carlo settings
x		For hierarchical and complex designs, identification of the appropriate level for tests and full reporting of outcomes
x		Estimates of effect sizes (e.g. Cohen's *d*, Pearson's *r*), indicating how they were calculated

Our web collection on statistics for biologists contains articles on many of the points above.

Software and code

Policy information about availability of computer code

Data collection	We used the free software Mesquite v.3.01 for coding morphological data.
Data analysis	We used the software TNT v. 1.1 for carrying out the phylogenetic analysis.

For manuscripts utilizing custom algorithms or software that are central to the research but not yet described in published literature, software must be made available to editors/reviewers. We strongly encourage code deposition in a community repository (e.g. GitHub). See the Nature Research guidelines for submitting code & software for further information.

Data

Policy information about availability of data

All manuscripts must include a data availability statement. This statement should provide the following information, where applicable:

- Accession codes, unique identifiers, or web links for publicly available datasets
- A list of figures that have associated raw data
- A description of any restrictions on data availability

All the data is provided in the manuscript and supplementary information files.

Field-specific reporting

Please select the one below that is the best fit for your research. If you are not sure, read the appropriate sections before making your selection.

- [ ] Life sciences
- [ ] Behavioural & social sciences
- [x] Ecological, evolutionary & environmental sciences

For a reference copy of the document with all sections, see nature.com/documents/nr-reporting-summary-flat.pdf

Ecological, evolutionary & environmental sciences study design

All studies must disclose on these points even when the disclosure is negative.

Study description	This study focuses on the description of two new species and a nesting site of titanosaurid sauropod dinosaurs. It includes a description and comparison of the species, as well as an analysis of their phylogenetic relationships. The result of both comparisons and phylogenetic data support the hypothesis of a close relationship between the Late Cretaceous dinosaur faunas of northern and southern South America
Research sample	The sample includes the new sauropod species Punatitan coughlini and Bravasaurus arrierosorum, as well as egg clutches, eggs and eggshell fragments, all recovered at the Quebrada de Santo Domingo locality. Punatitan consists of a partial skeleton (CRILAR-Pv 614). Bravasaurus consists of two specimens (CRILAR-Pv 612 and CRILAR-Pv 613). The oological material includes two partial egg clutches, an isolated egg, and many eggshell samples (CRILAR-Pv 620 and CRILAR-Pv 621). All these samples are curated at the Vertebrate Paleontology Collection of CRILAR, La Rioja, Argentina.
Sampling strategy	We collected the specimens reported here on fieldwork carried out between 2015 and 2019. During this period, we made 6 trips of 15-25 days each.
Data collection	We collect paleontological and geological data in the field and laboratory work. In the field, S. Rocher and L. Leuzinger measured orientation of the fossil-bearing strata and the remains found, with a geological compass. They also performed the sedimentological analysis and description. E. M. Hechenleitner prepared most of the specimens, using classical tools, and with A. G. Martinelli, L. E. Fiorelli and L. Salgado translated the anatomical information into the data set used in the phylogenetic analysis. E. M. Hechenleitner, L. Leuzinger, and A. G. Martinelli photographed the specimens. L. Leuzinger and L. E. Fiorelli prepared and measured the eggshell samples.
Timing and spatial scale	In the field, we collected the data continuously, over an area of about 1.5 square kilometers. In the lab, anatomical information was collected after specimen preparation.
Data exclusions	We did not exclude data.
Reproducibility	Information for carrying out the phylogenetic analysis (data set) is given in Supplementary Data 2
Randomization	It does not apply to the study of fossils such as those described in this manuscript.
Blinding	Blinding is not used in investigations of this type.

Did the study involve field work? ☒ Yes ☐ No

Field work, collection and transport

Field conditions	We conducted fieldwork in a desertic region of western La Rioja, at around 3200 m.a.s.l. The region is only accessible in the summer-fall season of the southern hemisphere, due to low temperatures.
Location	Quebrada Santo Domingo, close to Laguna Brava, Precordillera of La Rioja, NW, Argentina.
Access and import/export	The site is more than 60 km from the nearest town. The fieldwork involved a limited number of people (less than 10). The fossil quarries are located far from any road and can only be accessed on foot. Fieldwork requires high physical effort because of the rugged relief and the oxygen-depleted atmosphere of the region. The altitude and unstable weather guarantee days of extreme heat and nights with freezing temperatures.
Disturbance	The tasks carried out in this study produced no disturbance.

Reporting for specific materials, systems and methods

We require information from authors about some types of materials, experimental systems and methods used in many studies. Here, indicate whether each material, system or method listed is relevant to your study. If you are not sure if a list item applies to your research, read the appropriate section before selecting a response.

Materials & experimental systems

n/a	Involved in the study	
☒	☐	Antibodies
☒	☐	Eukaryotic cell lines
☐	☒	Palaeontology
☒	☐	Animals and other organisms
☒	☐	Human research participants
☒	☐	Clinical data

Methods

n/a	Involved in the study	
☒	☐	ChIP-seq
☒	☐	Flow cytometry
☒	☐	MRI-based neuroimaging

Palaeontology

Specimen provenance

The species and nesting site described here come from the Ciénaga del Río Huaco Formation, at Quebrada Santo Domingo, La Rioja, Argentina.

Specimen deposition

The specimens are deposited in the Vertebrate Paleontology Collection of CRILAR, La Rioja.

Dating methods

We do not apply dating methods in this work. The data on the age of the fossil units come from other investigations.

☐ Tick this box to confirm that the raw and calibrated dates are available in the paper or in Supplementary Information.
